# Prevalence of depressive symptoms and symptoms of post-traumatic stress disorder among newly arrived refugees and asylum seekers in Germany: systematic review and meta-analysis

**DOI:** 10.1192/bjo.2021.54

**Published:** 2021-05-03

**Authors:** Andreas Hoell, Eirini Kourmpeli, Hans Joachim Salize, Andreas Heinz, Frank Padberg, Ute Habel, Inge Kamp-Becker, Edgar Höhne, Kerem Böge, Malek Bajbouj

**Affiliations:** Department of Psychiatry and Psychotherapy, Central Institute of Mental Health, Medical Faculty Mannheim, University Heidelberg, Germany; Department of Psychiatry and Psychotherapy, Central Institute of Mental Health, Medical Faculty Mannheim, University Heidelberg, Germany; Department of Psychiatry and Psychotherapy, Central Institute of Mental Health, Medical Faculty Mannheim, University Heidelberg, Germany; Department of Psychiatry and Psychotherapy, Campus Charité Mitte, Charité Universitätsmedizin Berlin, Germany; Department of Psychiatry and Psychotherapy, Campus Innenstadt, Clinic of the Ludwig-Maximilians-University (LMU) Munich, Germany; Department of Psychiatry, Psychotherapy and Psychosomatics, RWTH Aachen University, Germany; Department of Child and Adolescent Psychiatry, Psychosomatics and Psychotherapy, Faculty of Human Medicine, Philipps-University Marburg, Germany; Department of Child and Adolescent Psychiatry, Psychosomatics and Psychotherapy, Faculty of Human Medicine, Philipps-University Marburg, Germany; Department of Psychiatry and Psychotherapy, Campus Benjamin Franklin, Charité Universitätsmedizin, Germany; Department of Psychiatry and Psychotherapy, Campus Benjamin Franklin, Charité Universitätsmedizin, Germany

**Keywords:** Refugees, asylum seeker, depression, stress disorders, meta-analysis

## Abstract

**Background:**

In total numbers, Germany has faced the largest number of refugees and asylum seekers (RAS) in Europe in the past decade. Although a considerable proportion have experienced traumatic and stressful life events, there is no systematic review to date examining the prevalence of depressive symptoms and post-traumatic stress disorder (PTSD) symptoms in RAS in Germany.

**Aims:**

To calculate the prevalence of depressive symptoms and PTSD symptoms in the general population of RAS living in Germany after the year 2000 and explore the impact of study- and participant-related characteristics on prevalence estimates.

**Method:**

We systematically searched PubMed, CINAHL, PsycINFO, PSYNDEX, Academic Search Complete, Science Direct and Web of Science from January 2000 to May 2020 to identify articles reporting prevalence of depressive symptoms and PTSD in RAS in Germany (PROSPERO registration number: CRD42020182796).

**Results:**

In total, 31 different surveys met inclusion criteria with 20 surveys reporting prevalence estimates of depressive symptoms and 25 surveys symptoms of PTSD. Based on screening tools, the pooled prevalence estimate of PTSD symptoms was 29.9% (95% CI 20.8–38.7%) and of depressive symptoms 39.8% (95% CI 29.8–50.1%). Heterogeneity was large within and between subgroups. In multivariate meta-regressions on depressive symptoms, heterogeneity was largely explained by survey period, length of field period and study quality.

**Conclusions:**

Prevalence rates of depressive symptoms and PTSD symptoms in RAS are notably large. They exceed the prevalence in the general German population. As a result of high heterogeneity, however, pooled prevalence rates should be interpreted with caution.

## Background

Worldwide nearly 80 million people have been forcibly displaced by violence, crime, persecution and violations of human rights by the end of 2019.^1^ As an immediate consequence, Europe and Germany have been witnessing the largest wave of migration in their recent history with a peak in the years 2015 and 2016. In total numbers, Germany had the highest number of first-time applicants for asylum with 441 899 and 722 370 in 2015 and 2016 respectively^2^ and it is the leading country for first-time applicants worldwide in the past decade.^1^ Likewise, Germany registered the largest number of asylum claims from unaccompanied children in Europe in the past decade.^1^

## Mental health issues

A considerable number of refugees and asylum seekers (RAS) has experienced traumatic and stressful life events often associated with mental and physical suffering.^3,4^ This can hamper the integration process into the host country, for example it can inhibit language acquisition, may lead to exclusion from active social participation, and can increase the risk of impulsive and delinquent behaviour.^5^ Therefore, it seems reasonable to assume that RAS belong to one of the most vulnerable groups concerning mental health problems. However, RAS experience multiple barriers to adequate mental healthcare for institutional, cultural and linguistic reasons. The asylum application process and its legal underpinnings may have a negative impact on adequate access to mental health services.^6^ Likewise, precarious living conditions in the host country, insecure residency status and legal restrictions to fulfil basic needs regarding employment or study may aggravate symptoms of depression and post-traumatic stress disorder (PTSD) in RAS.^7,8^ Intercultural expertise and communication in mental health services are still insufficient.^9^ For policy planning and for the provision of adequate mental healthcare, it is important to be aware of the prevalence of depression and PTSD, particularly after the recent peak of migration.

## The existing literature

At present, there are some international systematic reviews and meta-analyses on PTSD and depression in RAS.^10–15^ Prevalence rates in predominant representative samples of RAS vary widely from 14%^11^ to 44%^14^ for depression and 23%^11^ to 35%^13^ for PTSD. Although prevalence ranges are partly attributable to different methodological approaches, all reviews equally concluded that host country conditions could affect the level of symptoms.^10–15^ Most reviews included studies years before the peak of arriving refugees to Europe in 2015 and 2016.^12,14,15^ More recent reviews with meta-analyses were undertaken solely in children and adolescents,^11,13^ or in the adult population.^10^ Moreover, most of these reviews report on a range of host countries that have different legal procedure and reception systems.

## Aims

We wanted to review and estimate the prevalence of depressive symptoms and symptoms of PTSD, in the general population of RAS in Germany regardless of the age of RAS. Simultaneously, we wanted to include recent surveys done on RAS who arrived Germany in 2015 and later. We concentrated on two common mental disorders, although it is likely that generalised anxiety disorders (GAD) also frequently occur in RAS. However, rates of GAD in RAS in Germany are infrequently reported. Against this background, we aimed to:

- investigate the prevalence of depressive symptoms and PTSD symptoms in RAS in Germany;
- explore the impact of study characteristics and participant-related characteristics on prevalence estimates via subgroup analyses; and
- compare the prevalence estimates in the general population of RAS with the prevalence in the general German population.

## Method

### Literature search strategy and selection criteria

We adhered to the Preferred Reporting Items of Systematic Reviews and Meta-Analyses (PRISMA) statements (Supplementary Data 1 PRISMA checklist available at https://doi.org/10.1192/bjo.2021.54).^16^ We registered the protocol with PROSPERO (number CRD42020182796). First, we systematically searched international literature databases PubMed, CINAHL, PsycINFO, PSYNDEX, Academic Search Complete, Science Direct and Web of Science to identify relevant articles on 30 April 2020. We used a combination of Medical Subject Headings (MeSH) and text-based search terms. The MeSH terms included ‘mental health’ or ‘mental disorder’ and ‘refugees’ or ‘asylum-seeker’. The full-text search terms substantiated the disorder (i.e. depression and PTSD) and place (Germany). We used filters to identify papers written in English or German published between 1 January 2000 and 30 April 2020. Exemplary search strings are provided in Supplementary Tables 1–4.

We included articles and reports that assessed the prevalence of depressive symptoms, PTSD symptoms or both in samples of RAS living in Germany without limits on sample size, age and country of origin. We defined asylum seekers according to the European Migration Network as people who entered a host country to seek protection and whose claims are either awaiting preparation, submission or adjudication.^17^ Likewise, we defined refugees as people who, either owing to a well-founded fear of persecution for reasons of religion, nationality, political opinion or membership of a particular social group, are outside the country of nationality and are unable or, owing to such fear, are unwilling to avail themselves of the protection of that country.^17^ However, in practice, in some German surveys, the term ‘refugee’ is loosely applied and may also comprise people whose applications for asylum had been rejected, and a temporary suspension of deportation has been issued, or people with subsidiary protection.

We included population-based samples living in Germany where symptoms were assessed via international diagnostic schemes, i.e. the DSM or ICD, or via screening tools. We excluded:

(a) samples recruited from psychiatric or mental health settings and samples taken from claims data of health insurance companies;
(b) samples that had lived in Germany on average for 6 years or longer;
(c) studies/surveys that reported aggregated measures of mental disorders (e.g. the Refugee Health Screener).

The records of the systematic literature search were transferred to Endnote X9.3 (Clarivate Analytics, Philadelphia, Pennsylvania, USA). One author (A.Ho.) undertook the preliminary check for duplicates, other languages and types of publication. To finalise the literature database search, two authors (A.Ho. and E.K.) checked the remaining titles and abstracts for eligibility and in cases of disagreement, a third author (H.J.S.) was consulted.

One author (E.K.) did the additional search of the reference lists of eligible titles from the literature database search. Another author (A.Ho.) performed the online search for reports provided in German via Open Grey, and Google using the same search terms and time filters as mentioned above. We contacted 11 first authors of studies and surveys for additional information regarding methodology and data and received eight responses. In two cases, we received non-published papers or reports and, in another case, the authors provided an additional analysis upon request. In one case, we got exclusive access to the raw data. This survey also contains the largest sample of refugees so far in Germany, the so-called refugee panel as part of the socioeconomic panel with *n* = 4465 refugees.^18^ We used the raw data-sets to calculate the prevalence rate based on PHQ-2 values using a cut-off from the literature.^19^

### Data extraction

Using a standardised form two authors (E.K. and A.Ho.) started to extract the prevalence of mental disorders and participant and study characteristics on 28 May 2020. The participant characteristics included mean age, country of origin, percentage of included males, percentage of included unaccompanied minor refugees (UMR), accommodation and average duration of residency in Germany.

Study characteristics contained study design, sampling procedure, diagnostic or screening tool with applied cut-off value, base population, sample size, geographic location and field period of the survey. The (estimated) size of the base population and the sample size were used to calculate response rates. In some cases, our reported prevalence differed from the original, because we considered the total sample size including those participants with incomplete data. We listed each study under the first authors’ last name and year of publication.

### Quality assessment/risk of bias

Methodological quality was assessed following an adapted quality-assessment approach for observational studies originally developed by Barendregt and Doi.^20^ Our template comprised six elements, each valued from zero to two or three points. The sum of values, i.e. the quality score, had a possible range from 0 to 14 points. We evaluated the provision of clear and comprehensive descriptions of the sample (definition of the basic population, observation period, target population and included sample). In addition, we assessed the survey/field method, i.e. community survey, street survey or register-based method and the case ascertainment, i.e. the use of diagnostic systems or screening tools. Furthermore, we rated the type of administration of measurements, i.e. interviews or self-administered questionnaires and the representativeness of the surveys. Finally, we assessed the type of prevalence measure. For specification and details, see Supplementary Data 2 and Supplementary Table 5. Two reviewers (A.Ho. and E.K.) critically appraised each study independently and condensed the information into a final risk of bias rating for each individual survey using three distinct categories: low (12–14 points), moderate (10–11 points) and high (0–9 points). Disagreement was resolved through discussion.

### Data analysis

We anticipated heterogeneity because of between-study variations in study design, sample population and methods.^21^ Therefore, we performed a meta-analysis of prevalence using the method of inverse variance heterogeneity based on the quality-effects model.^22^ The explanatory power of each study (i.e. study weight) is corrected for study size, the amount of random variation and study quality. The study quality scores served to calculate the quality index, a rescaled score with values from zeros to one. We divided each rescaled score by the score of the highest scoring study in order to achieve relative quality ranks. We adopted the study quality-effect model to calculate pooled prevalence and 95% CIs for depressive symptoms and PTSD symptoms.^22^ We transformed the prevalence using the Freeman–Tukey double-arcsine transformation^23^ to address the challenges of proportions closer to the confidence limits and invariance instability.^21^ The results were visualised via forest plots.

We assessed and quantified heterogeneity between studies via Cochran's *Q* and *I*^2^ statistics. The quality-effects model corrects the Cochran's *Q* for over dispersion based on its χ^2^ statistics. This results in broader confidence intervals in case of heterogeneity but leaves the study weights unaffected.^21^ *I*^2^ statistics describe the proportion of variation across included surveys caused by heterogeneity rather than by chance. We used the general recommendations to categorise *I*^2^-values: 25% as low, 50% as moderate and 75% as high heterogeneity.^24^ Likewise, we computed τ as the dispersion of the true effect sizes between surveys in term of the scale of effect size.

We examined publication bias by visual inspection of funnel plots and Doi plots. Asymmetry in funnel plots were depicted funnel shaped with standard error on the vertical axis and double-arcsine prevalence on the horizontal axis.^25^ The Doi plot is a sensitive approach to detect asymmetry by direct comparison of effect magnitude measures and appeared as a mountain plot with the most precise surveys on top and midpoint of the graph.^26^ The associated Luis Furuya–Kanamori (LFK) index is a quantitative measure of Doi plot asymmetry with values around zero. Current interpretations of values: within + 1 or – 1 for no asymmetry, > + 1 or – 1 but lower than + 2 or – 2 minor asymmetry, and + 2 or – 2 and larger for major asymmetry.^26^ To identify outliers, we performed sensitivity analyses. We evaluated the influence of each included survey on the pooled prevalence estimate. Furthermore, we inspected the Doi plot asymmetry and the LFK index of the remaining surveys. Additional quality-effects models were performed without significant outliers.

We investigated potential sources of heterogeneity using subgroup analyses and meta-regressions. At first, we differentiated between surveys that applied diagnostic instruments and surveys using screening tools. We calculated and reported prevalence estimates separately. In subsequent subgroup analyses, we considered surveys that applied screening tools because they represented the vast majority of included surveys.

We dummy-coded variables on study characteristics: field period (surveys conducted within 2015–2016 versus surveys conducted before or afterwards); sampling procedure (representative, i.e., national surveys versus non-representative samples); survey quality (high versus moderate/low) according to the quality assessment; and sample size (sufficiently large versus insufficient). We conducted a sample size analysis to estimate the adequacy of sample sizes of included surveys to produce reliable prevalence estimates. We used the formula for sample size calculation from Naing et al^27^ and considered *n* = 325 participants as a sufficiently large sample. Likewise, we compared dummy-coded participant characteristics. Thus, we compared surveys in children/adolescents versus surveys in adults; surveys with predominantly male participants (more than nine out of ten male participants in a sample) versus mixed gender. We compared country of origin (predominantly from the Middle East, i.e. more than nine out of ten participants in a sample versus mixed country of origin); and average duration of residency (lower than 6 months versus 6 or more months in Germany). It was not possible to conduct subgroup analyses on UMR as stated in the protocol, because we did not identify sufficient surveys.

We performed a restricted maximum-likelihood random effect model (REML) with random intercept and fixed slopes. We used the metaReg macro by Wilson for SPSS on back-transformed prevalence estimates. In the crude model, we included each subgroup variable and in the multivariate model, we included the variables with the highest differences between categories. All regressions were adjusted for study quality. We performed all analyses with IBM SPSS Statistics for Windows, Version 25 (IBM Corporation, Armonk, NY, USA) and MetaXL for Windows, Version 5.3 (EpiGear International Pty Ltd, Sunrise Beach, Queensland, Australia).

## Results

### Review of selected studies

**Fig. 1:**
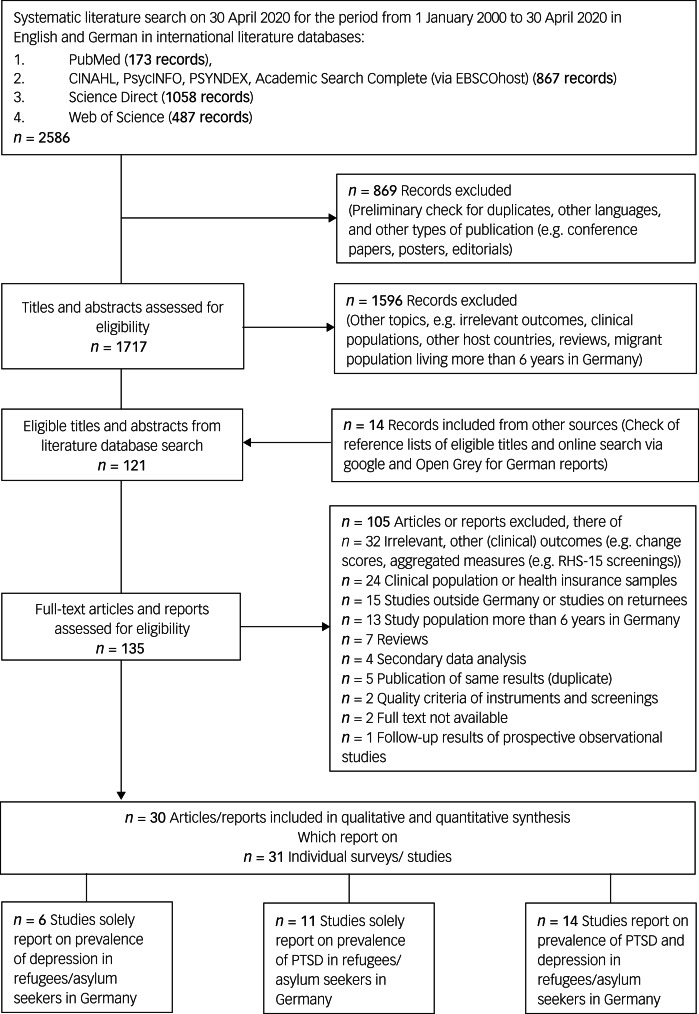
Search strategy and review process. PTSD, Post-traumatic stress disorder.

The systematic literature search provided 2586 records including duplicates (Fig. 1). After the removal of duplicates, other languages than English or German, and other types of publication, we found 1717 records eligible to be screened for inclusion criteria. We selected 121 articles for full-text review. The agreement between raters was almost perfect (*κ* = 0.955). The hand search of the reference list and the online search for grey literature provided 14 additional records. In total, we checked the eligibility of 135 full-text records. Of these, 30 articles describing 31 mutually exclusive studies/surveys satisfied our inclusion criteria.^28–57^

In total *n* = 12 002 RAS were surveyed. Six surveys solely reported on prevalence of depressive symptoms including *n* = 8066 RAS.^29–31,37,44,51^ Eleven surveys reported solely on prevalence of PTSD symptoms including *n* = 910 refugees.^32,33,36,39,42,43,50,53–55^ Both disorders were evaluated in 14 surveys including *n* = 3026 refugees.^28,34,35,38,40,41,45–49,52,56,57^ The sample sizes in surveys on prevalence of PTSD symptoms in RAS were primarily small (40% with a sample size less than 100 RAS), whereas only 15% of surveys of prevalence of depressive symptoms in RAS had a sample size less than 100 participants. A sufficient sample size according to the formula of Naing et al^27^ applied to the conditions of this review (i.e. *n* > 325) was fulfilled by 8% of surveys on prevalence of PTSD symptoms and 30% of surveys on prevalence of depressive symptoms.

In addition, the risk of bias was disparately larger in surveys on the prevalence of PTSD symptoms than in surveys on the prevalence of depressive symptoms. Seven surveys on prevalence of PTSD symptoms showed a high risk, *k* *=* 15 surveys a moderate risk and 3 surveys a low risk of bias. On the contrary, only two surveys on the prevalence of depressive symptoms showed a high risk of bias. Five surveys were classified a low risk and the majority of surveys a moderate risk of bias (*k* *=* 13).

In total, 42% of surveys were conducted during the years with the highest numbers of refugees arriving in Germany (2015–2016), whereas 29% of the surveys took place before as well as after these years. The participants were recruited from local refugee facilities (51.6%), the registry of the federal agency of migration and refugees (12.9%), by word-of-mouth recommendation in Arabic-speaking communities or mosques (9.7%), from care facilities owned by charities or youth welfare offices (9.7%), from the federal labour office/the job centre (6.5%) and from educational institutions or schools (6.5%). One survey did not specify the recruitment method.

The surveys were conducted in all regions of Germany, with the majority conducted in Southern Germany (45.2%) followed by Middle Germany (19.4%) and Northern Germany (16.1%). Five surveys (16.1%) aimed to cover all Federal states of Germany. For one survey, the location was unknown. Most included surveys draw convenience samples (52%), whereas 29% of included surveys used a full census of small areas such as collective accommodation centres and initial reception centres. Representative samples at a national or at least municipal level were drawn in 19% of included surveys. Survey characteristics and characteristics on participants are shown in detail in Supplementary Data 3 and Supplementary Table 6.

### Surveys on prevalence of PTSD symptoms

Data on prevalence of symptoms of PTSD could be retrieved from *k* *=* 25 surveys including *n* = 3936 RAS. The average age of participants in PTSD-prevalence surveys was 23.7 years (s.d. = 9.5). The majority were men (68.3%). Most participants originated from the Middle East (73.1%) and their average length of residence in Germany was 15.3 months (s.d. = 16.5). A rather low number of surveys (32%) were conducted in children and adolescents. Only 12% of the surveys included UMR. Two surveys (8%) used a diagnostic instrument as well as a screening tool^47,48^, while 24% used solely diagnostic instruments according to DSM or ICD and 68% screening tools with specific cut-offs to assess prevalence rates of PTSD symptoms.

**Fig. 2:**
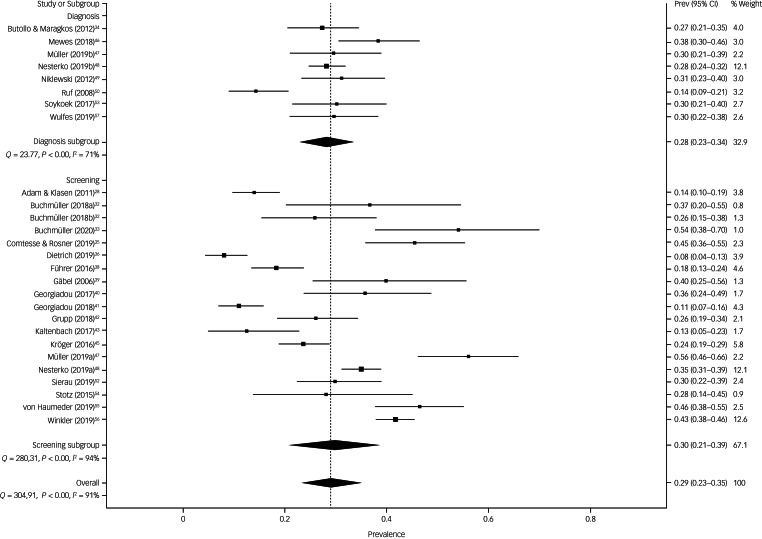
The prevalence estimate of symptoms of post-traumatic stress disorder (PTSD) in surveys using diagnostic instruments and screening tools. Prev, prevalence.

The prevalence estimate of symptoms of PTSD was 28.1% (95% CI 22.9–33.6%) in surveys using diagnostic instruments and 29.9% (95% CI 20.8–38.7%) in surveys using screening tools (Fig. 2). There was substantial heterogeneity between surveys (surveys with diagnostics: *Q* = 23.8, d.f. = 7, *P* < 0.001, *I*^2^ = 70.5%, τ = 0.122 and surveys with screenings: *Q* = 280.3, d.f. = 18, *P* < 0.001, *I*^2^ = 93.6%, τ = 0.298). The funnel plot of prevalence by sample size does not support the likelihood of publication bias (Supplementary Fig. 1). Likewise, the Doi plot and the LFK-index of 0.03 showed no asymmetry, verifying the absence of bias (Supplementary Fig. 2).

Sensitivity analyses did not provide evidence for outliers (Supplementary Table 7). For the final subgroup analyses, we included all 19 surveys using screening tools. Subgroup analyses based on the quality-effect model, yielded some differences in prevalence (see Table 1). Pooled prevalence estimates of PTSD symptoms in RAS were lower in surveys where participants resided for a longer period in Germany (24.5%) rather than shorter periods (33.3%), in surveys conducted primarily in male participants in comparison with surveys with mixed gender (40.6% *v.* 28.5%) and in surveys with shorter field periods (21.1%) compared with surveys with longer field periods (35.7%).

**Table 1:** Subgroup analysis on surveys using screening tools for the prevalence of symptoms of post-traumatic stress disorder (PTSD) and depression in refugees and asylum seekers in Germany

Methodological factor and subgroup categories	Quality-effect model symptoms of PTSD			Quality-effect model symptoms of depression		
	Pooled prevalence, % (95% CI)^a^	*I^2^* (%)	*Q-s*tatistic (d.f.)	Pooled prevalence, % (95% CI)^a^	*I^2^* (%)	*Q-s*tatistic (d.f.)
Survey period						
Before and after the refugee crisis	31.5 (17.9–46.1)	93.9	147.9 (9)	31.9 (24.1–40.0)	89.6	77.3 (8)
Refugee crisis	28.0 (15.2–41.7)	93.8	129.1 (8)	42.3 (29.6–55.1)	98.2	337.0 (6)
Study design^b^						
No representative samples	28.5 (20.1–37.3)^c^	91.1	179.8 (16)	33.5 (25.3–42.0)	91.2	113.4 (10)
Representative samples	33.3 (0.0–83.0)^c^	98.9	93.4 (1)	41.7 (28.8–55.0)	98.8	320.9 (4)
Risk of bias^d^						
Moderate to high	28.9 (19.2–39.0)^c^	93.5	263.2 (17)	43.1 (30.5–56.0)	93.3	165.4 (11)
Low	35.0 (31.1–38.9)^c^	–	– (0)	35.2 (17.6–53.9)	98.8	258.3 (3)
Mean residency in Germany^e^						
7 months and above	24.5 (13.8–36.0)	93.8	192.9 (12)	41.7 (35.9–47.5)	84.7	65.4 (10)
Up to 6 months	33.3 (23.0–44.1)	93.3	59.5 (4)	34.4 (14.4–55.8)	99.0	382.7 (4)
Field period						
7 months and above	35.7 (25.7–45.9)	89.8	88.2 (8)	42.6 (32.2–53.2)	97.3	221.7 (6)
Up to 6 months	21.1 (11.9–31.1)	92.3	116.2 (9)	30.2 (18.0–43.2)	94.6	148.9 (8)
Gender						
Male gender	40.6 (25.2–56.5)	82.1	16.7 (3)	33.6 (27.4–39.9)	39.0	3.3 (2)
Mixed gender	28.5 (19.1–38.3)	94.4	247.8 (14)	40.1 (29.5–51.0)	97.5	476.3 (12)
Region of origin						
Predominantly Middle East	23.6 (9.8–39.1)	94.5	126.6 (7)	36.2 (17.9–55.6)	97.1	207.8 (6)
Mixed region of origin	31.6 (21.8–41.8)	92.8	139.1 (10)	41.8 (28.5–55.4)	96.7	244.3 (8)
Sample size^f^						
Insufficient sample size	24.1 (16.2–32.4)^c^	92.1	202.7(16)	38.3 (31.7–45.1)	85.0	59.8 (9)
Sufficient sample size	38.4 (31.9–45.0)^c^	82.7	5.8 (1)	40.0 (27.1–53.4)	98.8	424.0 (5)
Age group						
Adult	30.4 (20.4–40.8)	94.3	209.2 (12)	40.1 (29.3–51.1)	97.5	481.8 (12)
Children/adolescents	28.1 (12.4–45.4)	91.6	59.8 (5)	35.2 (30.6–39.8)	0.0	1.1 (2)
Screening tool^g^						
PHQ-9	–	–	–	30.7 (21.8–40.0)	89.7	67.7 (7)
All other screeners	–	–	–	41.8 (30.1–53.7)	98.0	344.0 (7)

*I*^2^, proportion of observed variance; *Q*-statistic, weighted sum of square difference between observed effect and average effect; PHQ, Patient Health Questionnaire.

a. Based on the back-transformed double-arcsine prevalence estimate using the quality-effect model.

b. Representativeness was defined according to catchment area: national or multisite surveys were considered as (broadly) representative and single-site surveys or convenience samples as not representative.

c. Unable to perform meta-regression because of the low number of surveys in a subgroup category (*k* ≤ 2).

d. Risk of bias was defined according to the quality assessment of low risk (>11 points) and moderate to high risk (≤11 points) see Supplementary Data 2.

e. One survey with no data in quality-effect model on symptoms of PTSD.

f. Sufficient sample size was defined using the formula by Naing et al^27^ (see methods), i.e. *n* ≥ 325 participants.

g. Differentiation between screening tools was possible for depressive symptoms only: PHQ-9 with cut-off greater than 9 against all other screeners see Supplementary Table Supplementary Table 5.

Additionally, we carried out meta-regressions using the REML method adjusted for study quality to explore the impact of heterogeneity owing to survey characteristics. We were not able to perform meta-regressions for risk of bias, study design and sample size because of the insufficient number of surveys in associated subgroup categories. None of the remaining subgroup comparisons provided β-coefficients significantly different from zero.

### Surveys on prevalence of depressive symptoms

Data on prevalence of symptoms of depression in RAS could be retrieved from *k* *=* 20 surveys including *n* = 11 092 RAS. The average age of participants was 28.6 years (s.d. = 5.7). The majority of participants were male (73.5%), and came from the Middle East (74.8%). The average length of residence in Germany was 14.2 months (s.d. = 15.3). Only 15% of the surveys were conducted in children and adolescents with only one surveying in UMR. In total, 20% of surveys applied a diagnostic instrument according to DSM or ICD to assess prevalence rates of depressive symptoms, 40% used the screening instrument PHQ-9 and 40% used any other screening tool.

**Fig. 3:**
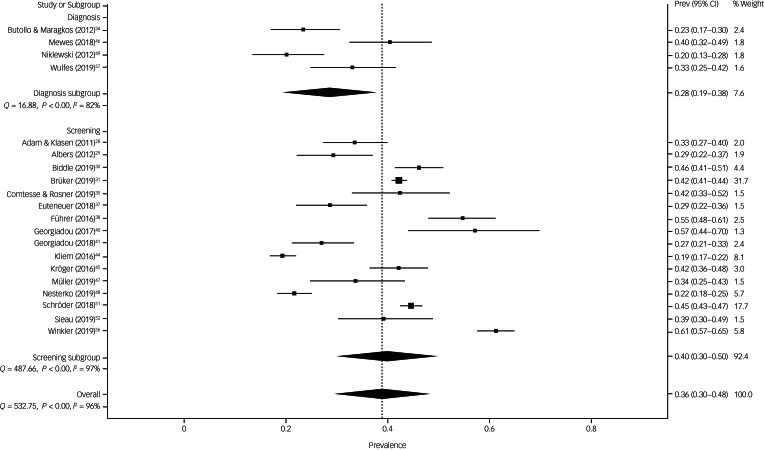
The prevalence estimate of symptoms of depression in surveys using diagnostic instruments and screening tools. Prev, prevalence.

The prevalence estimate of symptoms of depression was 28.4% (95% CI 19.4–37.8%) in surveys using diagnostic instruments and 39.8% (95% CI 29.8–50.1%) in surveys using screening tools (Fig. 3). The difference between survey methods was significant (12.2%, 95% CI 2.5–21.9%, *P* = 0.014). There was substantial heterogeneity within surveys (surveys with diagnostics: *Q* = 16.9, d.f. = 3, *P* < 0.001, *I*^2^ = 82.2%, τ = 0.184 and surveys with screenings: *Q* = 487.7, d.f. = 15, *P* < 0.001, *I*^2^ = 96.9%, τ = 0.241).

The funnel plot showed some asymmetry in the dispersion of studies (Supplementary Fig. 3). Likewise, the Doi plot showed a minor asymmetry indicating publication bias, which was confirmed by the LFK-index of −1.80 (Supplementary Fig. 4). Sensitivity analyses revealed no irregularities in pooled prevalence estimates and corresponding 95% confidence limits (Supplementary Table 8).

All 16 surveys that used screening tools were included in the final subgroup analyses (Table 1). The pooled prevalence estimates of depressive symptoms were greater in surveys conducted in the years 2015–2016 (44.3%, 95% CI 36.8–51.8%) compared with surveys conducted before and afterwards (32.1%, 95% CI 26.0–38.3%). The coefficient in the crude meta-regression was significantly different from zero: β = 12.4, 95% CI 1.6–23.2, *P* = 0.024. In addition, we found higher prevalence estimates in surveys with a higher risk of bias in comparison with surveys with low risk (43.1% *v.* 35.2%), in surveys using longer field periods (42.6%) than surveys with shorter field periods (30.2%), and in surveys using any other screening tool (41.8%) than the PHQ-9 (30.7%).

The associated β-coefficient for theses crude REML meta-regressions were all insignificant (Table 2). Table 2 also depicts coefficients for the multiple meta-regression with these four subgroup variables. The β-coefficients varied slightly in three variables and dropped largely for the subgroup variable screening tool. Although risk of bias, field period and applied screening tool remained insignificant single predictors of heterogeneity, in the multivariate model all variables accounted for 52.4% of heterogeneity in prevalence estimates (*R*^2^ = 0.5241).

**Table 2:** Meta-regression on surveys using screening tools for the prevalence of symptoms of depression in refugees and asylum seekers in Germany

Subgroup categories (*k*)^a^	Meta-regression analysis (REML) – crude model^b^			Meta-regression analysis (REML) - multivariate^c^		
	Coefficient (s.e.)^d^	95% CI	*P*	Coefficient (s.e.)^d^	95% CI	*P*
Survey period						
Before and after the refugee crisis (*k* = 9)	(reference)			(reference)		
Refugee crisis (*k* = 7)	12.40 (5.50)	(1.61 to 23.19)	0.024	12.05 (5.42)	(1.43 to 22.67)	0.026
Study design^e^						
No representative samples (*k* = 11)	(reference)					
Representative samples (*k* = 5)	5.85 (6.72)	(−7.68 to 18.65)	0.414			
Risk of bias^f^						
Moderate to high (*k* = 12)	(reference)			(reference)		
Low (*k* = 4)	−8.02 (7.04)	(−21.83 to 5.78)	0.255	−8.55 (5.06)	(−18.46 to 1.36)	0.091
Mean residency in Germany						
7 months and above (*k* = 11)	(reference)					
Up to 6 months (*k* = 5)	−5.98 (7.19)	(−20.07 to 8.10)	0.405			
Field period						
7 months and above (*k* = 7)	(reference)			(reference)		
Up to 6 months (*k* = 9)	−8.07 (6.05)	(−19.93 to 3.80)	0.183	−10.77 (5.99)	(−22.51 to 0.98)	0.072
Gender						
Male gender (*k* = 3)	(reference)					
Mixed gender (*k* = 13)	6.24 (8.00)	(−9.43 to 21.91)	0.435			
Region of origin						
Predominantly Middle East (*k* = 7)	(reference)					
Mixed region of origin (*k* = 9)	3.58 (6.35)	(−8.87 to 16.04)	0.573			
Sample size^g^						
Insufficient sample size (*k* = 10)	(reference)					
Sufficient sample size (*k* = 6)	0.41 (6.58)	(−12.49 to 13.32)	0.950			
Age group						
Adult (*k* = 13)	(reference)					
Children/adolescents (*k* = 3)	−4.25 (8.09)	(−20.11 to 11.60)	0.599			
Screening tool						
All other screeners (*k* = 8)	(reference)			(reference)		
PHQ-9 (*k* = 8)	−10.70 (5.75)	(−21.98 to 0.58)	0.063	−3.08 (5.63)	(−14.12 to 7.96)	0.585

REML, restricted maximum likelihood method; PHQ, Patient Health Questionnaire.

a. Number of surveys in each subgroup.

b. Crude model: meta-regression of the variable of interest using REML method adjusted for study quality.

c. Using survey period, risk of bias and length of field period in multivariate regressions with REML method adjusted for study quality. This model provided *R*^2^ of 0.5241.

d. Coefficient based on back-transformed double-arcsine group estimate.

e. Representativeness was defined according to catchment area: national or multisite surveys were considered as (broadly) representative and single-site surveys or convenience samples as not representative.

f. Risk of bias was defined according to the quality assessment of low risk (>11 points) and moderate to high risk (≤11 points) see Supplementary Data 2.

g. Sufficient sample size was defined using the formula 1 (see methods), i.e. *n* ≥ 325 participants.

## Discussion

### Main findings

We identified 30 studies yielding overall 20 prevalence estimates for depressive symptoms and 25 for PTSD symptoms in RAS in Germany after the year 2000. The number of studies using screening tools outnumbered studies using diagnostic instruments. We found significantly higher prevalence estimates for depressive symptoms using screening tools (39.8%) in comparison with diagnostic instruments (28.4%), whereas survey methods provided comparable prevalence estimates for symptoms of PTSD (28.1% *v.* 29.9%).

We found substantial heterogeneity across studies using screening tools for depressive symptoms and symptoms of PTSD in RAS in Germany. Heterogeneity in surveys on rates of depressive symptoms could partly be explained by the length of the field period, risk of bias and survey period. Thus, data acquisition periods that lasted longer than 6 months provided higher prevalence estimates, and surveys with low risk of bias provided lower prevalence estimates. The largest contribution factor to heterogeneity was survey period. Surveys undertaken within the years 2015–2016 provided substantially higher prevalence estimates of depressive symptoms than surveys undertaken in the years before or afterwards. On the contrary, we were not able to explain heterogeneity in surveys using screening tools for PTSD symptoms by *a priori* selected subgroup variables. Although there were some differences on a descriptive level, meta-regressions revealed that these factors did not significantly contribute to heterogeneity.

### Comparison with international findings in RAS

We noted that recently published meta-analyses and systematic reviews using international data on the prevalence of depressive symptoms in adult RAS were roughly in accordance with our findings. Overall and consistent with our results, prevalence estimates of depressive symptoms in RAS were lower in in meta-analyses based solely on clinical interviews than in meta-analyses based on clinical interviews and screening tools. Thus, Lindert and colleagues found slightly higher pooled prevalence estimates (44%, 95% CI 27–62%)^14^ using a mixture of self-reported measures and clinician-administered interviews and Blackmore et al reported a pooled prevalence of 31.5% (95% CI 22.6–40.4%)^10^ using clinically validated diagnostic instruments only. In child and adolescent populations, we observed the same effect, although reported prevalence estimates of depression were lower than in adults. Blackmore et al included diagnostic instruments and found a pooled prevalence of 13.8%^11^ whereas Kien et al reported 20.7%^13^ when combining screening tools and diagnostic instruments.

Prevalence estimates of PTSD symptoms in RAS from international meta-analyses were equally divergent. In adult populations, Blackmore et al reported a similar pooled prevalence (31.5%)^10^ compared with our findings. In child and adolescent populations of RAS our findings were in between reported estimates by Blackmore et al (22.7%) based on clinical interviews^11^ and Kien et al (35.3%) based on clinical interviews and screening tools.^13^

Screening tools tend to overestimate prevalence rates,^8^ but there were even differences between culturally and clinically validated screening tools and screeners with currently unknown eligibility in RAS. Thus, the pooled prevalence estimate of depressive symptoms in RAS in Germany screened with the PHQ-9 equalled the prevalence estimate measured with clinical interviews on a descriptive level in our meta-analysis. The PHQ-9 is widely used in many languages and is recommended as a patient-reported outcome measure by the International Society for Pharmacoeconomics and Outcome Research.

Besides the use of different survey methods possible explanations for the variations in pooled estimates between meta-analyses were different periods of included studies, strategies to avoid risk of bias or to improve representativeness of estimates or to avoid heterogeneity between studies (i.e. different eligibility criteria such as sample sizes, identification of cases and inclusion of host countries). In addition, all the aforementioned meta-analyses applied random-effect models to synthesise prevalence estimates, whereas we used the quality-effects model with double-arcsine transformation. Thus, our confidence intervals were usually larger.^21^

Our meta-analysis included RAS of any age and we compared subgroups of adult samples (minimum mean age of 18 years) with children and adolescent samples (mean age less than 18 years). All other reviews focused either on adults or on children and adolescents. It seemed that prevalence estimates of depressive symptoms are lower in children and adolescents than in adult RAS. Our findings support this impression, although we could only identify a small number of studies that provided prevalence rates in children and adolescent RAS in Germany. Results on prevalence estimates of PTSD in RAS concerning differences between children/ adolescents and adults in international reviews including our review were inconclusive. We intended to perform subgroup analyses on accompanied versus UMR to link levels of psychological distress and mental health disorders to different circumstances during the flight and in the host country, where UMR go into the custody of Child and Youth Welfare Systems.^47^ However, we were not able to retrieve the necessary information.

All above-mentioned meta-analyses showed substantial heterogeneity. Beside methodological reasons, the authors related the high variability, inter alia, to length of stay in the host countries and the legal status of RAS. Recently published reviews by Giacco et al^3^ and Blackmore et al^10^ refer to a possible relationship between depression and length of displacement from the home country. Higher prevalence rates of depression were observable the longer RAS were displaced from the home country. Our results tended toward the same direction. These results might reveal insufficient social integration and inadequate access to mental health services for RAS that could increase rates of depressive symptoms in the long term.^3^ On the other hand, we found a tendency towards lower prevalence estimates of PTSD symptoms the longer RAS stayed in Germany. It could be argued that some RAS who experienced traumatic events heal spontaneously or without professional help in safe environments over time.^58^ It is equally likely that high prevalence rates measured shortly after arriving in Germany resulted from insensitive diagnostic or screening procedures, i.e. RAS who were false-positives due to ongoing context-specific distress that was indistinguishable from PTSD symptoms.^59,60^

### Comparison with the general German population

We compared the prevalence estimates of RAS found in our meta-analyses with representative surveys in the general German population from the literature. The 1-month prevalence of the full syndrome of PTSD for the adult German population is about 2.3% and additionally, the prevalence for the partial syndrome of PTSD is 2.7% measured with the Composite International Diagnostic Interviews measure and a PTSD checklist according to DSM-IV.^61^ The point prevalence of depressive symptoms for the general adult German population measured with the PHQ-8 is 10.1% (95% CI 9.6–10.7%)^62^ and for children and adolescents according to the Strength and Difficulties Questionnaire about 5.4% (95% CI 4.3–6.6%).^63^ The results found in our meta-analysis indicated that regardless of the survey method (screener or diagnostics) RAS in Germany seems to have higher prevalence estimates of PTSD symptoms and depressive symptoms compared with the general German population. Admittedly, these comparisons are a first indication of a disproportional distribution of prevalence rates of mental disorders in RAS and the general population in Western European countries. We were able to identify just one study that compared samples of RAS on certain predetermined sociodemographic characteristics and service use data with matched samples of individuals from the general population from the host country. This study from Australia found a more than three-fold risk of psychological distress and a more than four-fold risk of PTSD in a clinical refugee sample compared with a matched register-based Australian-born sample.^64^ Prevalence rates of depression in the general German population remain stable over time potentially concealing the effect of rising depression incidence due to improved prevention, mental healthcare and treatment benefits over time.^65^ However, RAS in Germany do not benefit from these measures and prevalence rates of mental disorders remain high or increase over time.^66^

### Meeting the mental health needs of RAS

Our meta-analysis comprised studies that included population-based samples of RAS only. These surveys followed an outreach approach to identify the estimated number of specific mental disorders in RAS who most likely had no or only limited contact with the German mental healthcare system by the time of investigation. Register-based studies on the basis of health insurance claim data^67^ or on billing data from local social welfare offices,^68^ who are responsible for medical needs during the time asylum seekers have no regular access to statutory health insurance, revealed strikingly low prevalence rates of mental disorders. For instance, depression was diagnosed in 4% of all cases according to claim data from a statutory health insurance^67^ and 2.6% of all cases according to billing data from a local social welfare office.^68^ We trace these low rates back to unidentified mental health needs in RAS possibly caused by low help-seeking behaviour, or inadequate access to mental health services. Thus, the proportion of asylum seekers with unmet mental health needs living in (initial) reception centres is large,^69^ and the utilisation of mental health services is generally low.^67^ However, we should be cautious not to interpret all high levels of alleged symptoms of PTSD and depression found in our review as signs of mental disorder, because we were not fully aware of the cultural and current living conditions of the RAS.^70^ Unfortunately, we were not able to retrieve comprehensive information related to the migration process from the included surveys of RAS in Germany.

It is beyond the scope of this review to discuss pre-, peri- and post-migration vulnerable conditions and the interplay with mental disorders, psychosocial well-being and resilience. We refer the reader to relevant reviews.^3,12,71–73^ In most host countries, RAS experience structural and sociocultural barriers to accessing healthcare resources. For instance, the German Asylum Seekers Benefits Act determines the access of asylum seekers to the mental healthcare system according to refugee status.^74^ Bureaucratic hurdles, language barriers, lack of funding of interpreters in in- and out-patient mental healthcare, lack of specialised mental healthcare services and/or extensively long waiting lists and divergent culture-specific expectations of RAS and healthcare professionals regarding care can hamper access.^75,76^

There is no routine path for psychological care and/or psychosocial support for RAS in the first weeks upon arrival in the host countries. On the one hand, mental health may deteriorate or distress increases with a negative impact on health outcomes and social participation in RAS. On the other hand, delayed diagnoses and interventions are often more expensive for the healthcare system. To reduce the individual suffering of RAS and the financial burden to the healthcare system, it is important to establish low threshold and culturally sensitive mental health services.^11,77^

Western European countries may learn from the mental health Gap Action Programme (mhGAP), a programme launched by the World Health Organization with the aim to provide effective mental health treatments for low- and middle-income countries where specialised mental health services are often lacking.^78^ This approach seeks task shifting to primary and community care, i.e. the transfer of a specific set of tasks or interventions usually performed by highly qualified professionals towards less qualified professions or supervised laypersons.^76^ This approach is promising for at least three reasons: (a) it is a low threshold strategy, (b) it is easy to implement in recommended stepped or collaborative care models (SCCM) for common mental disorders, and (c) it is likely to be cost-effective and simultaneously optimises resource allocation.

SCCM customises the intervention according to illness severity. Thus, at lower steps and for those with less severe symptoms counselling by social workers, refugee peer supporters and other non-medical staff could be feasible.^79^ Such an approach, which includes elements of e-mental health and peer support, has been used in a recently conducted study to promote Mental Health in Refugees and Asylum Seekers (MEHIRA) in Germany.^77^ Another low-threshold approach for RAS could be the Problem Management Plus programme, a scalable psychological intervention conducted by non-specialised helpers such as refugee peer supporters who were trained and supervised by mental health professionals.^79^ Refugee peer supporters are mentors from the same cultural background who are appropriately trained in access to the healthcare system of the host country. They could help RAS to cope with health concerns and perceived access barriers.^80^ Besides that, e-mental health solutions as stand-alone applications could be beneficial, too. Currently, modularised e-mental health solutions as self-help interventions are being tested in Syrian refugees in Germany for depression^81^ and PTSD.^82^

### Methodological considerations and limitations

Several limitations should be taken into account. First, the present meta-analysis concentrated on depressive symptoms and PTSD symptoms only. Admittedly, other mental disorders show high prevalence rates in RAS, such as psychosis, GAD or emotional and behavioural problems in children and adolescents,^10,11,13,83^ and studies on highly relevant disorders such as alcohol misuse and drug-related disorders are still lacking. However, most studies and surveys consider the individual mental suffering of RAS because of traumatising and stressful experiences. Thus, we considered primarily depressive symptoms and PTSD symptoms.

Second, we found substantial heterogeneity between studies in all regression models. Heterogeneity adversely affects interpretability of and conclusions drawn from pooled prevalence estimates. We performed subgroup analyses and meta-regressions, but substantial heterogeneity among included surveys on the prevalence of PTSD symptoms remained largely unexplained. The prevalence of PTSD symptoms among RAS could be affected by unexamined but observable factors such as exposure to war, violence and poverty, family status or educational attainment and post-migration factors such as social support, cultural adaption, access to education, challenges relating to legal procedures and socioeconomic status. However, we were not able to retrieve such information. Furthermore, it could be the case that our regression models were insufficient to explain within-survey variations in order to explain heterogeneity between surveys.^15^ The mutual influence of participant and study characteristics might be underestimated.

Third, we did not examine the criterion validity of individual screening tools used in RAS. Screenings differ considerably in execution procedures, quantity and grading of items and cut-off values leading to a great variance in precision. In addition, they mostly are not culturally validated. Consequently, screening tools do not only overestimate pathology but are also unable to catch culturally diverse characteristics of symptomatology, i.e. transcultural phenomena.^13^ Therefore, it is not clear whether screeners measure mental disorder or emotional distress.^60^ We included some surveys that used ultra-short screeners with different cut-offs in our analyses, thereof one survey with an extensively large weight.^31^ However, in sensitivity analyses, and additional analyses where we omitted the survey with the largest sample size, the results were nearly the same (data not shown).

Fourth, most of the included studies reporting prevalence of PTSD applied DSM-IV diagnostic criteria. These criteria have been criticised for limited recognition of cultural perspectives.^84^ Therefore, the intercultural application of a Western psychiatric framework with a heterogeneous group of displaced people experiencing stressful and traumatic life events might be inaccurate. Fifth, it was not entirely possible to create satisfactory subgroup categories for gender and country of origin. There is currently a lack of surveys reporting on female RAS or gender-specific information in surveys is not provided. The same is true for country of origin. Sixth, most included studies consisted of convenience samples or were recruited at single sites. This might limit the generalisation of our results. However, our subgroup analysis on study design indicated no significant differences in prevalence estimates between studies with representative and non-representative samples. Despite large differences in prevalence estimates of depressive symptoms and PTSD symptoms among RAS because of heterogeneity, meta-analyses provide useful information for public health policymakers. Instead of relying on singular surveys, meta-analyses provide differentiated considerations and reduce the possibility of erroneous decisions.

### Implications

Our meta-analysis confirms the initial assumption that RAS show high prevalence rates of depressive symptoms and PTSD symptoms. The prevalence seemed to be higher than in the general population of the host country. However, results should be handled cautiously because they were mostly based on screening tools that were not culturally adapted and validated. Nevertheless, both distress and mental disorders can affect the functional capacity and the social integration of RAS negatively.

It is important to establish low threshold and culturally relevant mental health services to facilitate the integration process, to reduce language barriers and counteract chronic courses of mental disorders. This includes: (a) task shifting, (b) the use of properly trained and supervised healthcare mentors from the same cultural background, (c) the promotion of culturally sensitive specialised services, and (d) the use of e-mental health solutions. Against a background of adequate resource allocation in mental healthcare, there is the opportunity for stepped and collaborative care models for RAS.

## Data Availability

Data are available upon request from the corresponding author.

## Supplementary material

For supplementary material accompanying this paper visit http://dx.doi.org/10.1192/bjo.2021.54. click here to view supplementary material
