# Supplementary material for: Prevalence of depressive symptoms and symptoms of post-traumatic stress disorder among newly arrived refugees and asylum seekers in Germany: systematic review and meta-analysis
Source: BJPsych Open. 2021 May 3;7(3):e93. doi: 10.1192/bjo.2021.54 (PMC8142547; doi:10.1192/bjo.2021.54)
Supplement: Supplementary file 1 [file bjosup.zip › S2056472421000545sup006.docx]

**Supplementary Data 2** Specification of Quality Assessment Variables to estimate Risk of Bias


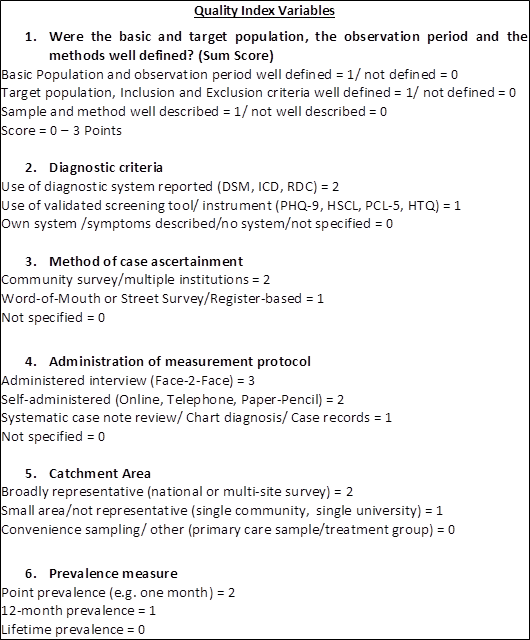


**Search strings for systematic review on prevalence of depression and PTSD in refugees and asylum seekers in Germany**

**Supplementary Table 1: PubMed/ Medline**

| 1 | Refugees [MeSH Terms] |
| --- | --- |
| 2 | Refugee* [Text Word] |
| 3 | Asylum?seek* [Text Word] |
| 4 | Forced Migr* [Text Word] |
| 5 | Or/1-4 |
| 6 | Mental Health [MeSH Term] |
| 7 | Mental Disorders [MeSH Term] |
| 8 | Mental Disorder* [Text Word] |
| 9 | Mental Illness* [Text Word] |
| 10 | Depressive Disorders [MeSH Term] |
| 11 | Depression [MeSH Term] |
| 12 | Depress* [Text Word] |
| 13 | Mood Disord* [Text Word] |
| 14 | Stress Disorder, Post Traumatic [MeSH Term] |
| 15 | Posttraumatic Stress Disorder [MeSH Term] |
| 16 | PTSD* [text word] |
| 17 | Post?traumatic Stress Disorder* [Text Word] |
| 18 | Posttraumatic Stress Disorder* [Text Word] |
| 19 | Or/6-18 |
| 20 | Germany [MeSH Term] |
| 21 | German* [Text Word] |
| 22 | Or/20-21 |
| 23 | 5 And 19 And 22 |
| 24 | Limit 23 - Filters: Publication date from 2000/01/01 to 2020/12/31; English; German |

**Supplementary Table 2: Ebsco – including Academic Search Complete, CINAHL, PsycInfo, PSYNDEX**

| S1 | Refugees OR Asylum Seekers [Search Modes: Find all my search terms] |
| --- | --- |
| S2 | Asylum Seeker* [Search Modes: Find all my search terms] |
| S3 | Refugee* [Search Modes: Find all my search terms] |
| S4 | Forced Migration [Search Modes: Find all my search terms] |
| S5 | S1 OR S2 OR S3 OR S4 |
| S6 | Mental Health OR Mental Illness OR Mental Disorder [Search Modes: Find all my search terms] |
| S7 | Stress |
| S8 | Stress Disorders, Post-traumatic |
| S9 | Depression OR Depressive Disorder OR Depressive Symptoms OR Major Depressives Disorder [Search Modes: Find all my search terms] |
| S10 | Post Traumatic Stress Disorder [Search Modes: Find all my search terms] |
| S11 | PTSD OR Posttraumatic Stress Disorder OR Post-traumatic Stress Disorder [Search Modes: Find all my search terms] |
| S12 | Mood Disorders [Search Modes: Find all my search terms] |
| S13 | S6 OR S7 OR S8 OR S9 OR S10 OR S11 OR S12 |
| S14 | Germany OR German |
| S15 | S5 AND S13 AND S14 |
| S16 | Limit S15 - Limiters: Published Date: 20000101-20201231; English; German |

**Supplementary Table 3: Science Direct**

| 1 | Refugees (Find this term] |
| --- | --- |
| 2 | Asylum Seeker (Find this term] |
| 3 | 1 OR 2 |
| 4 | “Mental Health” (Find this term] |
| 5 | “Mental Disorder” (Find this term] |
| 6 | Depression (Find this term] |
| 7 | Depressive (Find this term] |
| 8 | Post Traumatic (Find this term] |
| 9 | Posttraumatic (Find this term] |
| 10 | 4 OR 5 OR 6 OR 7 OR 8 OR 9 |
| 11 | Germany |
| 12 | 3 AND 10 AND 11 |
| 13 | Limit 12 – Refined by Years: 2000-2020; Article Type: Review articles AND Research articles |

**Supplementary Table 4: Web of Science/ Social Sciences Citation Index (SSCI)**

| #1 | TS=(Refugee*) |
| --- | --- |
| #2 | TS=(Asylum Seeker*) |
| #3 | ALL=(Refugee*) |
| #4 | ALL=(Asylum seeker OR asylum-seeker) |
| #5 | ALL=(Forced Migr*) |
| #6 | #1 OR #2 OR #3 OR #4 OR #5 |
| #7 | TS=(Mental Health) |
| #8 | TS=(Mental Disorder) |
| #9 | TS=(Mental Illness) |
| #10 | TS=(Depression OR Depressive Disorder) |
| #11 | TS=( Post-traumatic Stress* OR Posttraumatic Stress* OR Post Traumatic Stress*) |
| #12 | ALL=( Depress* OR Stress Disorder) |
| #13 | ALL=( Mood Disord*) |
| #14 | ALL=(PTSD OR Post Traumatic OR Posttraumatic OR Post-traumatic) |
| #15 | #7 OR #8 OR #9 OR #10 OR #11 OR #12 OR #13 OR #14 |
| #16 | TS=(Germany OR German) |
| #17 | ALL=(German*) |
| #18 | #16 OR #17 |
| #18 | #6 AND #15 AND #18 |
| #19 | Limit #18 – Indexes=SSCI Timespan 2000-2020 AND Language: (English OR German) |

**Supplementary Table 5** Quality assessment of included studies in meta-analyses on depressive symptoms and PTSD symptoms in refugees and asylum seekers in Germany

| # | Studies | Dep. | PTSD | **Quality Index Variables** | | | | | | Total  (Max: 14) | 13 |
| --- | --- | --- | --- | --- | --- | --- | --- | --- | --- | --- | --- |
|  |  |  |  | 1 | 2 | 3 | 4 | 5 | 6 |  |  |
| 1 | Adam, 2011 | 1 | 1 | 2 | 1 | 2 | 2 | 0 | 2 | 9 | 0.692 |
| 2 | Albers, 2012 | 1 | 0 | 3 | 1 | 2 | 2 | 1 | 2 | 11 | 0.846 |
| 3 | Biddle, 2019 | 1 | 0 | 3 | 1 | 2 | 2 | 2 | 2 | 12 | 0.923 |
| 4 | Brücker, 2019 | 1 | 0 | 1 | 1 | 2 | 2 | 2 | 2 | 10 | 0.769 |
| 5a | Buchmüller, 2018a | 0 | 1 | 1 | 1 | 1 | 2 | 0 | 2 | 7 | 0.538 |
| 5b | Buchmüller, 2018b | 0 | 1 | 2 | 1 | 1 | 2 | 0 | 2 | 8 | 0.615 |
| 6 | Buchmüller, 2020 | 0 | 1 | 2 | 1 | 1 | 2 | 0 | 2 | 8 | 0.615 |
| 7 | Butollo, 2012 | 1 | 1 | 3 | 2 | 2 | 3 | 1 | 2 | 13 | 1 |
| 8 | Comtesse, 2019 | 1 | 1 | 2 | 1 | 2 | 3 | 0 | 2 | 10 | 0.769 |
| 9 | Dietrich, 2019 | 0 | 1 | 3 | 1 | 1 | 2 | 2 | 2 | 11 | 0.846 |
| 10 | Euteneuer, 2018 | 1 | 0 | 1 | 1 | 2 | 2 | 0 | 2 | 8 | 0.615 |
| 11 | Führer, 2016 | 1 | 1 | 3 | 1 | 2 | 2 | 1 | 2 | 11 | 0.846 |
| 12 | Gäbel, 2006 | 0 | 1 | 1 | 2 | 2 | 3 | 0 | 2 | 10 | 0.769 |
| 13 | Georgiadou, 2017 | 1 | 1 | 3 | 1 | 2 | 2 | 1 | 2 | 11 | 0.846 |
| 14 | Georgiadou, 2018 | 1 | 1 | 3 | 1 | 2 | 2 | 1 | 2 | 11 | 0.846 |
| 15 | Grupp, 2018 | 0 | 1 | 1 | 1 | 2 | 2 | 0 | 2 | 8 | 0.615 |
| 16 | Kaltenbach, 2017 | 0 | 1 | 2 | 1 | 2 | 3 | 1 | 2 | 11 | 0.846 |
| 17 | Kliem, 2016 | 1 | 0 | 3 | 1 | 2 | 2 | 2 | 2 | 12 | 0.923 |
| 18 | Kröger, 2016 | 1 | 1 | 2 | 1 | 2 | 3 | 1 | 2 | 11 | 0.846 |
| 19 | Mewes, 2018 | 1 | 1 | 2 | 2 | 1 | 3 | 0 | 2 | 10 | 0.769 |
| 20 | Müller, 2019 | 1 | 1 | 2 | 1 | 2 | 3 | 0 | 2 | 10 | 0.769 |
| 21 | Nesterko, 2019 | 1 | 1 | 3 | 1 | 2 | 3 | 1 | 2 | 12 | 0.923 |
| 22 | Niklewski, 2012 | 1 | 1 | 1 | 2 | 2 | 3 | 1 | 2 | 11 | 0.846 |
| 23 | Ruf, 2008 | 0 | 1 | 2 | 2 | 2 | 3 | 0 | 2 | 11 | 0.846 |
| 24 | Schröder, 2018 | 1 | 0 | 3 | 1 | 2 | 2 | 2 | 2 | 12 | 0.923 |
| 25 | Sierau, 2019 | 1 | 1 | 2 | 1 | 2 | 3 | 0 | 2 | 10 | 0.769 |
| 26 | Soykoek, 2017 | 0 | 1 | 2 | 2 | 2 | 3 | 1 | 2 | 12 | 0.923 |
| 27 | Stotz, 2015 | 0 | 1 | 0 | 1 | 2 | 3 | 0 | 2 | 8 | 0.615 |
| 28 | von Haumeder, 2019 | 0 | 1 | 1 | 1 | 2 | 3 | 0 | 2 | 9 | 0.692 |
| 29 | Winkler, 2019 | 1 | 1 | 2 | 1 | 2 | 2 | 2 | 2 | 11 | 0.846 |
| 30 | Wulfes, 2019 | 1 | 1 | 0 | 2 | 2 | 3 | 1 | 2 | 10 | 0.769 |

*Legend.* Column # depicts running numbers of included studies. Column Dep. = symptoms of depression and column PTSD = symptoms of Post-Traumatic Stress Disorder indicate if studies reported on symptoms (=1) or not (=0). Blue columns contain the ratings on Quality Index Variables that are specified in figure 1 of this Supplement. Column “Total” contains the sum score of Quality Index Variables with a possible range from zero to 14. The last column contains relative quality ranks (Division of each score by the score of the highest scoring study to achieve values between zeros to one). Traffic lights display risk of bias: green = low risk of bias, yellow = moderate risk of bias, red = high risk of bias.

**Supplementary Data 3 – List of included studies in the qualitative and quantitative synthesis (in alphabetic order)**

Adam H, Klasen F. [Trauma and reconciliation: Child refugees in Hamburg]. Trauma & Gewalt. 2011; 5(4): 356-69.

Albers HM. [Living conditions and mental health of residents in a collective accommodation centre for asylum seekers in Würzburg]. Medizinische Fakultät: 116. Würzburg: Medizinische Fakultät der Julius-Maximilians-Universität Würzburg, 2012.

Biddle L, Menold N, Bentner M, Nöst S, Jahn R, Ziegler S, et al. Health monitoring among asylum seekers and refugees: a state-wide, cross-sectional, population-based study in Germany. Emerg Themes Epidemiol. 2019; 16(1): N.PAG-N.PAG.

Brücker H, Croisier J, Kosyakova Y, Kröger H, Pietrantuono G, Rother N, et al. [Language skills and employment rate of refugees in Germany improving with time] DIW Wochenbericht 4/2019: 56-70. Berlin: DIW Berlin — Deutsches Institut für Wirtschaftsforschung e. V., 2019.

Buchmüller T, Lembcke H, Busch J, Kumsta R, Leyendecker B. Exploring mental health status and syndrome patterns among young refugee children in Germany. Front Psychiatry. 2018; 9.

Buchmüller T, Lembcke H, Busch J, Kumsta R, Wolf OT, Leyendecker B. Exploring hair steroid concentrations in asylum seekers, internally displaced refugees, and immigrants. Stress. 2020.

Butollo W, Maragkos M. [Final report of the expert panel on asylum seekers: Detection of mental disorders in asylum seekers]. 100. München: Lehrstuhl Klinische Psychologie und Psychotherapie der Ludwig-Maximilians-Universität München, 2012.

Comtesse H, Rosner R. Prolonged grief disorder among asylum seekers in Germany: the influence of losses and residence status. Eur J Psychotraumatol. 2019; 10(1): 1591330.

Dietrich H, Al Ali R, Tagay S, Hebebrand J, Reissner V. Screening for posttraumatic stress disorder in young adult refugees from Syria and Iraq. Compr Psychiatry. 2019; 90: 73-81.

Euteneuer F, Schäfer SJ. Brief report: subjective social mobility and depressive symptoms in Syrian refugees to Germany. J Immigr Minor Health. 2018; 20(6): 1533-6.

Fuhrer A, Eichner F, Stang A. Morbidity of asylum seekers in a medium-sized German city. Eur J Epidemiol. 2016; 31(7): 703-6.

Gäbel U, Ruf M, Schauer M, Odenwald M, Neuner F. [Prevalence of posttraumatic stress disorder among asylum seekers in Germany and its detection in the application process for asylum]. Z Klin Psychol Psychother. 2006; 35(1): 12-20.

Georgiadou E, Morawa E, Erim Y. High manifestations of mental distress in arabic asylum seekers accommodated in collective centers for refugees in Germany (PSYNDEXshort). Int J Env Res Pub He. 2017; 14(6): 612-.

Georgiadou E, Zbidat A, Schmitt GM, Erim Y. Prevalence of mental distress among Syrian refugees with residence permission in Germany: A registry-based study (PSYNDEXshort). Front Psychiatry. 2018; 9: 393-.

Grupp F, Moro MR, Nater UM, Skandrani SM, Mewes R. "It's that route that makes us sick": Exploring lay beliefs about causes of post-traumatic stress disorder among sub-saharan African asylum seekers in Germany. Front Psychiatry. 2018; 9: 628.

Kaltenbach E, Härdtner E, Hermenau K, Schauer M, Elbert T. Efficient identification of mental health problems in refugees in Germany: The refugee health screener. Eur J Psychotraumatol. 2017; 8: 1389205-.

Kliem S, Mößle T, Klatt T, Fleischer S, Kudlacek D, Kröger C, et al. [Psychometric evaluation of an Arabic version of the PHQ-4 based on a representative survey of Syrian refugees]. PPmP. 2016; 66(9-10): 385-92.

Kröger C, Frantz I, Friel P, Heinrichs N. [Posttraumatic stress and depressive symptoms amongst asylum seekers. Screening in a state refugee reception center]. Psychother Psych Med. 2016; 66(9-10): 377-84.

Mewes R, Friele B, Bloemen E. Validation of the Protect Questionnaire: A tool to detect mental health problems in asylum seekers by non-health professionals. Torture 2018; 28(2): 56-71.

Müller LRF, Büter KP, Rosner R, Unterhitzenberger J. Mental health and associated stress factors in accompanied and unaccompanied refugee minors resettled in Germany: a cross-sectional study. Child Adolesc Ment Health. 2019; 13(1): N.PAG-N.PAG.

Nesterko Y, Jackle D, Friedrich M, Holzapfel L, Glaesmer H. Prevalence of post-traumatic stress disorder, depression and somatisation in recently arrived refugees in Germany: an epidemiological study. Epidemiol Psychiatr Sci. 2019; 29: e40.

Niklewski G, Richter K, Lehfeld H. [Report of the expert panel on asylum seekers: Detection of mental disorders in asylum seekers in Zirndorf]. 43. Nürnberg: Klinik für Psychiatrie und Psychotherapie, Klinikum Nürnberg, 2012.

Ruf M. [Traumatised refugee children in Germany epidemiology, treatment and neurocognitive as well as neurophysiological changes]. 374-. 2008.

Schröder H, Zok K, Faulbaum F. [Health of refugees in Germany - A survay on refugees from Syria, Iraq and Afghanistan]. WIdO Monitor. 2018; 15(1): 1-20.

Sierau S, Schneider E, Nesterko Y, von Klitzing K, Glaesmer H. [Mental health problems of unaccompanied young refugees in youth welfare institutions]. Psychiat Prax. 2019; 46(3): 135-40.

Soykoek S, Mall V, Nehring I, Henningsen P, Aberl S. Post-traumatic stress disorder in Syrian children of a German refugee camp. Lancet. 2017; 389(10072): 903-4.

Stotz SJ, Elbert T, Müller V, Schauer M. The relationship between trauma, shame, and guilt: findings from a community-based study of refugee minors in Germany. European Journal of Psychotraumatology. 2015; 6: 1-N.PAG.

von Haumeder A, Ghafoori B, Retailleau J. Psychological adaptation and posttraumatic stress disorder among Syrian refugees in Germany: a mixed-methods study investigating environmental factors. Eur J Psychotraumatol. 2019; 10(1): 1686801.

Winkler JG, Brandl EJ, Bretz HJ, Heinz A, Schouler-Ocak M. [The influence of residence status on psychiatric symptom load of asylum seekers in Germany]. Psychiat Prax. 2019; 46(4): 191-9.

Wulfes N, del Pozo MA, Buhr-Riehm B, Heinrichs N, Kroger C. Screening for posttraumatic stress disorder in refugees: Comparison of the diagnostic efficiency of two self-rating measures of posttraumatic stress disorder. J Trauma Stress. 2019; 32(1): 148-55.

**Supplementary Table 6**. Characteristics of included studies

| **First Author, Year** | **Region/ Federal state** | **Survey period** | **Study design/ Sample** | **Basic population/ Size** | **Sample size/ % Response rate** | **Country of Origin*** | **% Male** | **% UMR** | **Ave. Age, Years (SD)** | **Ave. Time in Germany, Months (SD)** | **Survey method** | **Instrument Depression** | **Instrument PTSD** | **Prev.**  **Depression** | **Prev. PTSD** |
| --- | --- | --- | --- | --- | --- | --- | --- | --- | --- | --- | --- | --- | --- | --- | --- |
| Adam, 2011 | Hamburg | 09.2002-06.2003 | cross-sectional/ convenience | All ASC 9-20 years going to one of 27 (out of 64 contacted) schools in Hamburg with > 9 ASC/ ? | 215/ ? | Afghanistan (80.9%), Bosnia (13.5%), Kosovo (5.6%) | 58.6 | 0 | 14.8 (2.1) | 68.4 (38.4) | Self-rated/ P&P/ Screening | DS-RSC, Cut-off >14 Pts | UCLA PTSD-RI, Cut-off >37 Pts | 33.5% | 14.0% |
| Albers, 2012 | Bavaria | 02.2011-07.2011 | cross-sectional/ single-site (full census) | all ASR >= 14 years living in one CAC in Würzburg/ n=450 | 140/ 31.1 | Somalia, Ethiopia, Afghanistan, Iraq | 59.0 | 0 | 29.2 (9.4) | 20.4 (30.0) | Self-rated/ P&P/ Screening | PHQ-9 Cut-off >=10 Pts | - | 29.5% | - |
| Biddle, 2019 | BW | 01.2018-12.2018 | cross-sectional/ random multi-site (representative on state level) | All ASR >= 18 years registered in BW and living in IRC or CAC in 2018 (65 units including n=2,346 people drawn from a total of estimated n=1,938 units including 70,634 people)/ n=987 eligible | 412/ 41.7 | Afghanistan (22.2%), Syria (14.4%), Gambia (10.0%), Iraq (9.7%), Iran (6.9%), Nigeria (5.3%), others (31.3%) | 68.8 | 0 | *Group*  18-25: 31.2%26-30: 17.9%31-35: 17.1%36-40: 14.2%41+: 19.7% | *Group*  0-6: 2.7%  7-12: 17.1% 13-15: 26.4%, 16-24: 43.5%, 25-36s: 10.2% | Self-rated/ P&P or Online/ Screening | PHQ-2, Cut-off >3 Pts | - | 46.0% | - |
| Brücker, 2019 | all Federal states | 2016 | Longitudinal survey (only first wave; survey year 2016) | Refugees who moved to Germany to seek asylum between 01.01.2013 to 01.31.2016 and were registered in the Central Register of Foreigners by 06.30.2016/ ? | 4465/? | Afghanistan (12.8%), Syria (49.0%), Iraq (12.9%), Eritrea (5.4%), other nationalities (18.6%), stateless (1.3%) | 73 | 0 | 34.0 (10.4) | 21.3 (12.3) | Self-rated/ F2F/ Screening | PHQ-2, Cut-off >2 Pts | - | 42.2% | - |
| Buchmüller, 2018a | NRW | 02.2017-11.2017 | cross-sectional/ convenience | Arabic-speaking AMR by word-of-mouth recommendation/ ? | 30/ ? | Syria (90%), Iraq (10%) | 49.0 | 0 | 3.7 (1.3) | 18.4 (9.7) | Observer-ratings/ F2F/ Screening | - | CBCL 1,5-5, Cut-off >9 Pts | - | 37.0% |
| Buchmüller, 2018b | NRW | 06.2017-07.2017 | cross-sectional/ convenience | AMR in educational institutions providing aid/? | 58/ ? | Syria (81%), Iraq (19%) | 43.0 | 0 | 3.6 (1.2) | 19.0 (5.7) | Observer-rating/ P&P/ Screening | - | CTRF 1,5-5, Cut-off >9 Pts | - | 26.0% |
| Buchmüller, 2020 | NRW | 05.2017-10.2018 | cross-sectional/ convenience | Word-of-mouth recommend-dation in Arabic-speaking mosques and local refugee camps/ ? | 37/? | Syria (100%) | 0 | 0 | 30.0 (5.3) | 20.6 (11.3) | Self-rated/P&P/ Screening | - | HTQ-PTSD according to DSM-IV, Cut-off >= 2.5 Pts | - | 57.1% |
| Butollo, 2012 | Bavaria | 10.2010-11.2011 | cross-sectional/ convenience | central appraisal office for all ASR arriving Munich (registered at IRC) in the survey period/ n=5,577 | 154/ 2.8 | Afghanistan (55.2%), Syria (12.3%), Sierra Leone (5.8%), China (5.8%), Turkey (5.2%), others (15.7%) | 68.8 | 0 | 27.8 (10) | 0.5 | Diagnosis by psychologist | M.I.N.I. according to DSM-IV and ICD-10 | PDS according to DSM-IV | 23.4% | 27.3% |
| Comtesse, 2019 | Bavaria | 12.2017-07.2018 | cross-sectional/ convenience | ASR aged 18 years or older from IRC and four decentralized CAC in Bavaria, Germany/ ? | 99/? | Arabic (45.4%), Kurdish (32.3%), Afghan (15.2%), Yazidi (2%), Persian (5.1%) | 67,7 | 0 | 30.1 (9.4) | 16.6 (12.9) | SSI/F2F/  Screening | PHQ-9, Cut-off >=10 Pts | PCL-5 according to DSM-5, Criteria: B, C, D and E | 42.4% | 45.5% |
| Dietrich, 2019 | all Federal states | 01.2016 | cross-sectional/ register-based (representative on national level) | all Syrians and Iraqis registered as unemployed in January 2016 in the unemployment register of the Federal Labour Office/ n=2.057 | 175/ 8.5 | Syria (84%), Iraq (16%) | 87.4 | 0 | 22.2 (2.2) | < 12 months: 77.7% | Self-rated/ CAWI or CATI/ Screening | - | ETI adapted, Cut-off >26 Pts, SSS-PSD | - | 8.0% |
| Euteneuer, 2018 | Hesse | 06.2017-07.2017 | cross-sectional/ convenience | ASR from Syria by word-of-mouth recommend-dation in and around Marburg/ ? | 164/ ? | Syria (100%) | 100 | 0 | 28.8 (8.2) | 11.4 (7.0) | Self-rated/ CAWI/ Screening | PHQ-9, Cut-off >=10 Pts | - | 28.7% | - |
| Fuehrer, 2016 | Saxony-Anhalt | 08.2015 | cross-sectional/ convenience | All ASR living in CAC in Halle (Saale)/ n=1,098 (560 eligible) | 214/ 38.2 | Syria (67.7%), Afghanistan (13.0%), Benin (5.1%), others (13.2%) | 85.0 | 0 | 29.2 (8.8) | 5.0 | Self-rated/ P&P/ Screening | HSCL-25 Cut-off >= 1.75 Pts | HTQ Part 4, Cut-off >= 2.5 Pts | 54.7% | 18.2% |
| Gäbel, 2006 | all Federal states | 09.2003-12.2003 | cross-sectional/ convenience | Selected cases of ASR from 16 individual decision-makers from 8 branch offices of the Federal Office for Migration and Refugees (Reutlingen, Karlsruhe, Wurzburg, Zirndorf, Munich,  Cologne, Dusseldorf Giessen)/ n=158 | 40/25.3 | Algeria (15%), Cameroon (2.5%), Nigeria (5%), Sudan (5%), Uganda (2.5%), Afghanistan (5%), India (10%), Iraq (12.5%), Iran (2.5%), PR China (2.5%), Ecuador (2.5%), Serbia-Montenegro (7.5%), Turkey (27.5%) | 92.5 | 0 | 26.6 (7.9) | 1.3 (0.7) | Diagnosis according to DSM-IV and ICD-10 | - | M-CIDI according to DSM-IV and ICD-10 | - | 40% |
| Georgiadou, 2017 | Bavaria | 08.2016-09.2016 | cross-sectional/ single-site (full census) | all Arabic-speaking ASR of the three CAC of the charity "German Samaritan Workers Union" in Erlangen/ n=80 (67 eligible) | 56/ 83.6 | Iraq (46.4%), Syria (33.9%), Palestine (8.9%), Syro-Palestine (5.4%), others (5.4%) | 64.3 | 0 | 27.2 (7.1) | 7.9 (3.7) | Self-rated/ P&P/ Screening | PHQ-9, Cut-off >=10 Pts | ETI adapted, Cut-off >26 Pts | 57.1% | 35.7% |
| Georgiadou, 2018 | Bavaria | 07.2017-12.2017 | cross-sectional/ register-based (representative on municipal level) | all Syrian ASR with a positive decision on asylum application registered in the job centre of Erlangen since 2014/ n=518 | 200/ 38.6 | Syria (100%) | 69.5 | 0 | 33.3 (10.5) | 23.3 (6.5) | Self-rated/ P&P/ Screening | PHQ-9, Cut-off >=10 Pts | ETI adapted, Cut-off >26 Pts | 27.0% | 11.4% |
| Grupp, 2018 | all Federal states | 04.2016-12.2016 | cross-sectional/  convenience and snowball sampling | ASR from Sub-Saharan Africa were recruited in accommodation centres with the help of civic refugee initiatives, from language courses for adult immigrants, and at religious and cultural gatherings/? | 119/? | Eritrea (34.5 %), Somalia (30.3 %), Ethiopia (5.9 %), Sudan (1.7 %), Cameroon (21.0 %), Nigeria (3.4 %), Togo (3.4 %) | 70.7 | 0 | 27.97 (7.8) | 24.0 (14.4) | P&P/ CAWI | - | PDS, Cut-off >= 21 Pts | - | 26% |
| Kaltenbach, 2017 | Southern Germany | over 3 weeks in 2016 | cross-sectional/ single-site (full census) | refugees aged >12 years living in a CAC in a rural area in southern Germany/ n=89 | 56/ 62.9 | Syria (58%), followed by Afghanistan (9%), Albania (8%), Kosovo (7%), Serbia (7%), Iraq (4%), Macedonia, Somalia, and Georgia (each 2%) | 64 | 0 | 28.8 (11.2) | 6.5 (3.0) | SSI/  F2F/  Screening | - | PCL-5 according to DSM-5 with Cut-off >=33 pts and sub-syndromal PTSD with Criterion A and criteria B – E from DSM-5 | - | 33.9% |
| Kliem, 2016 | Lower Saxony | 05.2015-08.2015 | cross-sectional/ register-based (representative on state level) | all Syrian ASR 18-50 years registered by the Foreigners' Registration Office in Lower Saxony during study period/ n=9,666 (1,554 eligible) | 864/ 55.6 | Syria (100%) | 68.0 | 0 | 31.5 (9.3) | 3 | Self-rated/ P&P/ Screening | PHQ-4, Cut-off >=3 Pts | - | 19.3% | - |
| Kröger, 2016 | Lower Saxony | 06.2015-08.2015 | cross-sectional/ single-site (full census) | all first-time ASR arriving at the IRC in Braunschweig in the summer of 2015/ n=1,737 | 280/ 16.1 | Balkans (20%), Middle East (14%), North Africa (43%), remaining Africa (23%) | 88.2 | 0 | 30.5 (9.8) | 3 | Self-rated/ F2F/ Screening | PHQ-9, Cut-off >=10 Pts | PDS-8, Cut-off >=12 Pts | 42.1% | 23.6% |
| Mewes, 2018 | Hesse | 02.2014-03.2015 | cross-sectional studies/convenience | ASR aged 18 years or older who had been living in Germany for a maximum of one year/? | 141/? | Iran (47.9%), Afganistan (16.0%), Syria (12.5%), Somalia (6.9%), Eritrea (5.6%), Algeria (2.1%), other countries/missing information (9.1%) | 67 | 0 | 31.9 (7.8) | 8.5 (7.5) | Diagnosis according to DSM-5/  Screening | SCID-1 | SCID-1 | 40.43% | 38.3% |
| Müller, 2019 | Bavaria | 04.2017-09.2017 | cross-sectional/ convenience | All ASC living in 19 out of 83 contacted care facilities for AMR and UMR and CAC in Bavaria/ ? | 98/ ? | Afghanistan (55.1%), Syria (14.3%), Eritrea (11.2%), Iraq (8.2%), others (11.2%) | 89.9 | 69.4 | 16.3 (1.7) | 21.5 (7.7) | Self-rated/ F2F/ Screening | HSCL-37A, Subscale Depression, Cut-off >= 33 Pts | CATS according to DSM-5, Cut-off >= 21 Pts | 33.7% | 56.1% (Screening), 29.6% (DSM-5 Diagnostics) |
| Nesterko, 2019 | Saxony | 05.2017-06.2018 | cross-sectional/ single-site (full census) | all registered ASR residing in IRC and CAC in Leipzig during the survey period/ n=1,316 | 569/ 43.2 | Cameroon (18.3%), Venezuela (16.9%), Syria (10.4%), Turkey (8.6%), Eritrea (8.2%), Nigeria (7.6%), others (30%) | 69.3 | 0 | 29.7 (8.8) | nearly 60% less than 7 days, approx. 20% 8-14 days | Self-rated/ F2F/ Screening | PHQ-9 Cut-off >=10 Pts | PCL-5, Cut-off >=33 Pts; and DSM-5 diagnostic critera | 21,70% | 34.9% (Screening), 28.2% (DSM-5 Diagnostics) |
| Niklewski, 2012 | Bavaria | 05.2011-01.2012 | cross-sectional, random single-site | All first-time ASR residing in IRC in Zirndorf during the survey period/ ? | 125 (random sample)/ ? | Iran (39.2%), Iraq (23.2%), Afghanistan (15.2%), Russia (8.8%), others (13.6%) | 65.6 | 0 | 31.7 (9.1) | ? | Diagnosis and Observer-rating by psychiatrist and psychologist | M.I.N.I. according to DSM-IV and ICD-10, BSI Subscale Depression, MADRS, WHO-5, Cut-off <13 Pts | M.I.N.I. according to DSM-IV and ICD-10, ETI | 19.7% | 31.2% |
| Ruf, 2010 | BW | 12.2003-12.2004 | cross-sectional/ convenience | AMR living in 13 CAC in BW/ ? | 104/ ? | Former Yugoslavia (29.8%), Turkey (15.4%), Iraq (12.5%), Syria (8.7%), Chechenia (7.7%), Iran (6.7%), others (19.2%) | 46.2 | 0 | 10.6 (2.6) | 43.0 (36.0) | Diagnosis by staff of outpatient department for refugees | - | UCLA PTSD-RI according to DSM-IV, M.I.N.I. KID | - | 19.2% |
| Schröder, 2018 | all Federal states | 05.22.2014-03.07.2018 | cross-sectional/ random multi-site (representative on national level) | all ASR from Syria, Iraq, and Afghanistan registered by the Federal Agency of Migration and Refugees between 2015 and May 2018/ n=825,226 | 2,021/ 0.2 | Syria (46.5%), Iraq (35.6%), Afghanistan (17.9%) | 67.1 | 0 | *Group* 18-29: 48.8%30-39: 27.3%40-49: 13.3%50+: 10.6% | During field phase: 61,7%; 2015: 19% und 2016: 19% | Self-rated/ CAWI or CAPI/ Screening | WHO-5, Cut-off <10.5 Pts | - | 44.6% | - |
| Sierau, 2019 | Saxony | 06.2017-08.2017 | cross-sectional/ convenience | UMR living in care facilities owned by the youth welfare office in Leipzig/ n=142 | 107/ 75.4 | Afghanistan (59.0%), Syria (34.3%), others (6.7%) | 100 | 100 | 17.3 (1.2) | 20.4 (7.2) | Self-rated/ F2Face/ Screening | PHQ-9 Cut-off >=10 Pts | PCL-5, Cut-off >=33 Pts | 40.9% | 30.5% |
| Soykoek, 2017 | Bavaria | 01.2014-06.2014 | cross-sectional/ single-site (full census) | AMR 0-14 years of Syrian origin registered at the IRC in Munich during the survey period/ n=198 | 96/ 48.9 | Syria (100%) | 54.2 | 0 | 7.2 (3.7) | 1.3 (1.4) | Diagnosis by CA psychiatrist and psychologist | - | *Infants (0-6 years):* PTBS-SSI according to DSM-IV, *CA (7-14 years):* Kinder-DIPS according to ICD-10 | - | 30.2% |
| Stotz, 2015 | BW | 09.2011-03.2012 | cross-sectional/ convenience | AMR and UMR living in care facilities owned by youth welfare offices/ ? | 32/ ? | Afghanistan, Iran, Iraq, Gambia, Nigeria, Sierra Leone, Kosovo, Serbia, Turkey | 100 | 56.3 | UMR: 17.4 (1.0), AMR: 15.6 (2.2) | 39.9 (49.8) | Self-rated/ F2Face/ Screening | - | UCLA PTSD according to DSM-IV | - | 28.1% |
| von Haumeder, 2019 | unknown | 11.2017-02.2018 | cross-sectional/ convenience | ASR from Syria >= 18 years receiving social support from one of 14 (out of 37 contacted) charities or municipal providers/ ? | 127/ ? | Syria (100%) | 66.1 | 0 | 31.9 (10.7) | 23.7 (10.4) | Self-rated/ F2Face/ Screening | - | PCL-5, Cut-off >=33 Pts | - | 46.5% |
| Winkler, 2019 | Berlin | 07.15.2015-03.01.2016 | cross-sectional, random multi-site (representative on state level) | All ASR >= 18 years living in CAC in Berlin during the survey period/ n=51,876 | 650/ 1.3 | Syria (36.9%), Afghanistan (14.9%), Iraq (8.5%), Albania (7.5%), Iran (6.3%), others (25.9%) | 74.8 | 0 | 30.6 (10.0) | 4.5 (6.3) | Self-rated/ P&P/ Screening | HSCL-25, Score >= 1,75 Pts | PDS according to ICD-10 supplemented by HTQ | 61.3% | 41.7% |
| Wulfes, 2019 | Lower Saxony | over the course of 5 days in 06.2016 and 02.2017 | cross-sectional/ convenience | ASR with a high prospect of a residency in Germany who lived in CAC in Braunschweig/? | 118/? | Syria (24.6%), Iraq (17.8%), Afghanistan (16.9%), Iran (16.1%), Sudan (11.0%), Turkey (5.1%), Somalia (2.5%), others (5.9%) | 64.4 | 0 | 32.9 (13.1) | ? | Diagnosis according to DSM-IV/  Screening | SCID-1 | SCID-1 | 33.1% | 29.7% |

*Only countries of origin with more than 5% participants. *Abbreviations:* AMR: accompanied minor refugees, approx.: approximately, ASC: Asylum-seeking Children and Adolescents, ASR: Asylum-seekers and/or Refugees, ave: average, BSI: Brief Symptom Inventory, BW: Baden-Wuerttemberg, CA: Children and Adolescents, CAC: collective accommodation centre, CATI: Computer-assisted Telephone Interview, CAWI: Computer-assisted Web Interview, CBCL: Childhood Behaviour Checklist, CATS: Child and Adolescent Trauma Screen, CTRF: Caregiver Teacher Report Form, DSM-IV: Diagnostic and Statistical Manual of Mental Disorder 4^th^ Edition, DSM: Diagnostic and Statistical Manual of Mental Disorder, DS-RSC: Depression Self-Rating Scale for Children, ETI: Essen Trauma Inventory, F2F: Face-to-Face, HSCL-25: Hopkins-Symptom Checklist 25 Item-Version, HSCL-37A: Hopkins-Symptom Checklist 37 Item Version for Adolescents, HTQ: Harvard Trauma Questionnaire, HTQ-PTSD: posttraumatic stress disorder scale of the Harvard Trauma Questionnaire, ICD: International Classification of Diseases, IRC: initial reception centre, Kinder-DIPS: Structured Diagnostic Interview for Mental Disorders for Children and Adolescents, MADRS: Montgomery-Asberg Depression Rating Scale, M-CIDI: Munich-Composite International Diagnostic Interview, M.I.N.I.: Mini International Neuropsychiatric Interview, M.I.N.I. KID: Mini International Neuropsychiatric Interview for Children and Adolescents, NRW: North Rhine-Westphalia, P&P: Paper-and-Pencil, PCL-5: Posttraumatic Stress Disorder Checklist for DSM-5, PHQ-2: Patient Health Questionnaire 2 Items Version, PHQ-4: Patient Health Questionnaire 4 Items Version, PHQ-9: Patient Health Questionnaire 9 Items Version, Prev.: Prevalence, PDS: Posttraumatic Diagnostic Scale, PDS-8: Posttraumatic Diagnostic Scale Short Version, Pts: Points, PTSD-SSI: Posttraumatic Stress Disorder Semi-structured Interview, SCID-1: The Structured Clinical Interview Axis I Disorders, SSI: Semi-structured Interview, SSS-PSD: Short Screening-Scale for Posttraumatic Stress Disorder, UCLA PTSD-RI: Childhood PTSD Reaction Index, UMR: unaccompanied minor refugees, WHO-5: World Health Organisation Five Well-Being Index.

**Sensitivity Analyses**

**Supplementary Table 7** Sensitivity analysis of studies reporting prevalence of symptoms of PTSD in refugees and asylum seekers in Germany

| **Excluded study** | **Pooled Prevalence** | **LCI 95%** | **HCI 95%** | **Cochran Q** | **p** | **I 2** | **I 2 LCI 95%** | **I 2 HCI 95%** |
| --- | --- | --- | --- | --- | --- | --- | --- | --- |
| Adam, 2011 | 0.306 | 0.237 | 0.377 | 263.058 | 0 | 91.257 | 88.265 | 93.486 |
| Buchmüller, 2018a | 0.295 | 0.227 | 0.366 | 295.163 | 0 | 92.208 | 89.644 | 94.136 |
| Buchmüller, 2018b | 0.296 | 0.227 | 0.227 | 295.618 | 0 | 92.220 | 89.662 | 94.145 |
| Buchmuller, 2020 | 0.294 | 0.227 | 0.363 | 286.494 | 0 | 91.972 | 89.304 | 93.974 |
| Butollo, 2012 | 0.297 | 0.226 | 0.370 | 295.572 | 0 | 92.218 | 89.660 | 94.144 |
| Comtesse, 2019 | 0.292 | 0.224 | 0.363 | 284.800 | 0 | 91.924 | 89.235 | 93.942 |
| Dietrich, 2019 | 0.308 | 0.244 | 0.374 | 236.670 | 0 | 90.282 | 86.834 | 92.827 |
| Führer, 2016 | 0.303 | 0.232 | 0.376 | 280.095 | 0 | 91.788 | 89.038 | 93.849 |
| Gäbel, 2006 | 0.295 | 0.227 | 0.365 | 293.889 | 0 | 92.174 | 89.596 | 94.113 |
| Georgiadou, 2017 | 0.295 | 0.226 | 0.366 | 294.884 | 0 | 92.200 | 89.634 | 94.131 |
| Georgiadou, 2018 | 0.307 | 0.241 | 0.376 | 249.681 | 0 | 90.788 | 87.579 | 93.168 |
| Grupp, 2018 | 0.297 | 0.227 | 0.370 | 295.260 | 0 | 92.210 | 89.648 | 94.138 |
| Kaltenbach, 2017 | 0.295 | 0.226 | 0.366 | 295.379 | 0 | 92.213 | 89.653 | 94.140 |
| Kröger, 2016 | 0.301 | 0.228 | 0.376 | 290.507 | 0 | 92.083 | 89.464 | 94.051 |
| Mewes, 2018 | 0.293 | 0.223 | 0.365 | 290.867 | 0 | 92.093 | 89.478 | 94.057 |
| Müller, 2019 | 0.290 | 0.224 | 0.357 | 266.262 | 0 | 91.362 | 88.418 | 93.557 |
| Nesterko, 2019 | 0.287 | 0.216 | 0.360 | 287.013 | 0 | 91.986 | 89.325 | 93.984 |
| Niklewski, 2012 | 0.295 | 0.225 | 0.368 | 295.754 | 0 | 92.223 | 89.667 | 94.147 |
| Ruf, 2008 | 0.302 | 0.233 | 0.373 | 276.224 | 0 | 91.673 | 88.871 | 93.770 |
| Sierau, 2019 | 0.296 | 0.226 | 0.368 | 295.930 | 0 | 92.228 | 89.674 | 94.150 |
| Soykoek, 2017 | 0.296 | 0.226 | 0.368 | 295.911 | 0 | 92.227 | 89.673 | 94.150 |
| Stotz, 2015 | 0.296 | 0.228 | 0.367 | 295.933 | 0 | 92.228 | 89.674 | 94.150 |
| von Haumeder, 2019 | 0.291 | 0.223 | 0.361 | 279.789 | 0 | 91.780 | 89.025 | 93.843 |
| Winkler, 2019 | 0.273 | 0.211 | 0.338 | 245.752 | 0 | 90.641 | 87.363 | 93.069 |
| Wulfes, 2019 | 0.296 | 0.226 | 0.368 | 295.940 | 0 | 92.228 | 89.674 | 94.151 |

Ρ (Rho) of zero indicates that there is no outlier concerning variations in pooled prevalence estimates and 95% confidence limits.

**Supplementary Table 8** Sensitivity analysis of studies reporting prevalence of symptoms of depression in refugees and asylum seekers in Germany

| **Excluded study** | **Pooled Prevalence** | **LCI 95%** | **HCI 95%** | **Cochran Q** | **ρ** | **I²** | **I² LCI 95%** | **I² HCI 95%** |
| --- | --- | --- | --- | --- | --- | --- | --- | --- |
| Adam, 2011 | 0.389 | 0.297 | 0.485 | 529.322 | 0 | 96.599 | 95.638 | 97.349 |
| Albers, 2012 | 0.390 | 0.299 | 0.485 | 526.239 | 0 | 96.580 | 95.611 | 97.335 |
| Biddle, 2019 | 0.384 | 0.290 | 0.483 | 525.216 | 0 | 96.573 | 95.601 | 97.330 |
| Brücker, 2019 | 0.374 | 0.276 | 0.477 | 510.700 | 0 | 96.475 | 95.466 | 97.260 |
| Butollo, 2012 | 0.393 | 0.298 | 0.493 | 513.841 | 0 | 96.497 | 95.496 | 97.275 |
| Comtesse, 2019 | 0.387 | 0.296 | 0.482 | 532.389 | 0 | 96.619 | 95.665 | 97.363 |
| Euteneuer, 2018 | 0.390 | 0.299 | 0.484 | 524.064 | 0 | 96.565 | 95.591 | 97.324 |
| Führer, 2016 | 0.384 | 0.293 | 0.478 | 512.626 | 0 | 96.489 | 95.485 | 97.269 |
| Georgiadou, 2017 | 0.385 | 0.296 | 0.479 | 525.750 | 0 | 96.576 | 95.606 | 97.332 |
| Georgiadou, 2018 | 0.391 | 0.300 | 0.486 | 518.373 | 0 | 96.528 | 95.539 | 97.297 |
| Kliem, 2016 | 0.407 | 0.326 | 0.491 | 343.433 | 0 | 94.759 | 93.032 | 96.058 |
| Kröger, 2016 | 0.387 | 0.294 | 0.484 | 531.940 | 0 | 96.616 | 95.661 | 97.361 |
| Mewes, 2018 | 0.387 | 0.296 | 0.483 | 532.694 | 0 | 96.621 | 95.668 | 97.364 |
| Müller, 2019 | 0.389 | 0.298 | 0.483 | 531.337 | 0 | 96.612 | 95.656 | 97.358 |
| Nesterko, 2019 | 0.399 | 0.310 | 0.491 | 440.346 | 0 | 95.912 | 94.679 | 96.860 |
| Niklewski, 2012 | 0.392 | 0.302 | 0.485 | 509.550 | 0 | 96.467 | 95.455 | 97.254 |
| Schröder, 2018 | 0.375 | 0.268 | 0.489 | 507.230 | 0 | 96.451 | 95.433 | 97.243 |
| Sierau, 2019 | 0.388 | 0.297 | 0.483 | 532.743 | 0 | 96.621 | 95.668 | 97.365 |
| Winkler, 2019 | 0.374 | 0.290 | 0.462 | 401.198 | 0 | 95.513 | 94.114 | 96.580 |
| Wulfes, 2019 | 0.389 | 0.298 | 0.484 | 530.636 | 0 | 96.608 | 95.650 | 97.355 |

Ρ (Rho) of zero indicates that there is no outlier concerning variations in pooled prevalence estimates and 95% confidence limits.
