# Supplementary figures and images for: Prevalence of depressive symptoms and symptoms of post-traumatic stress disorder among newly arrived refugees and asylum seekers in Germany: systematic review and meta-analysis
Source: BJPsych Open. 2021 May 3;7(3):e93. doi: 10.1192/bjo.2021.54 (PMC8142547; doi:10.1192/bjo.2021.54)

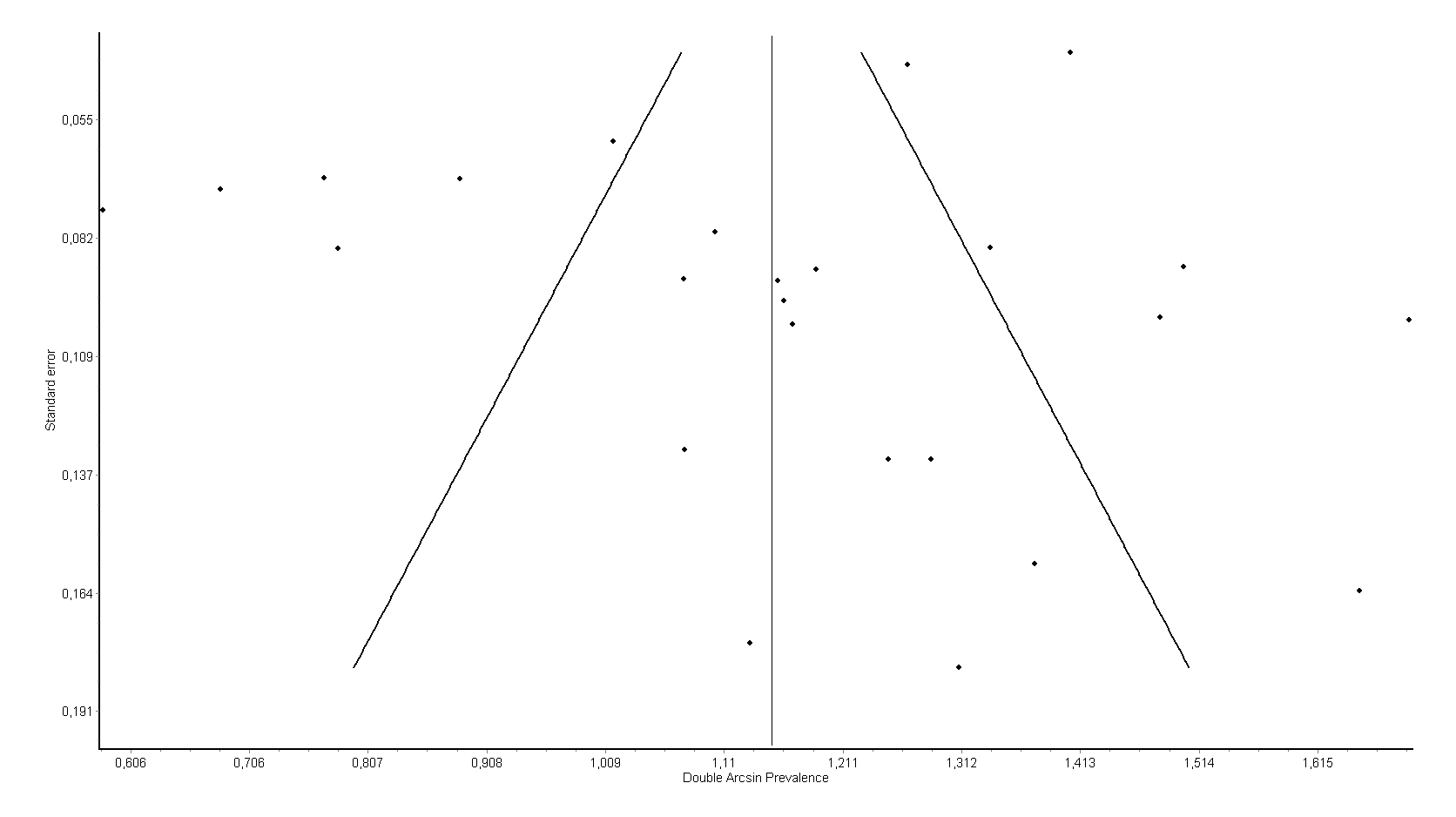

Supplement: Supplementary file 1 [file bjosup.zip › S2056472421000545sup001.tiff]

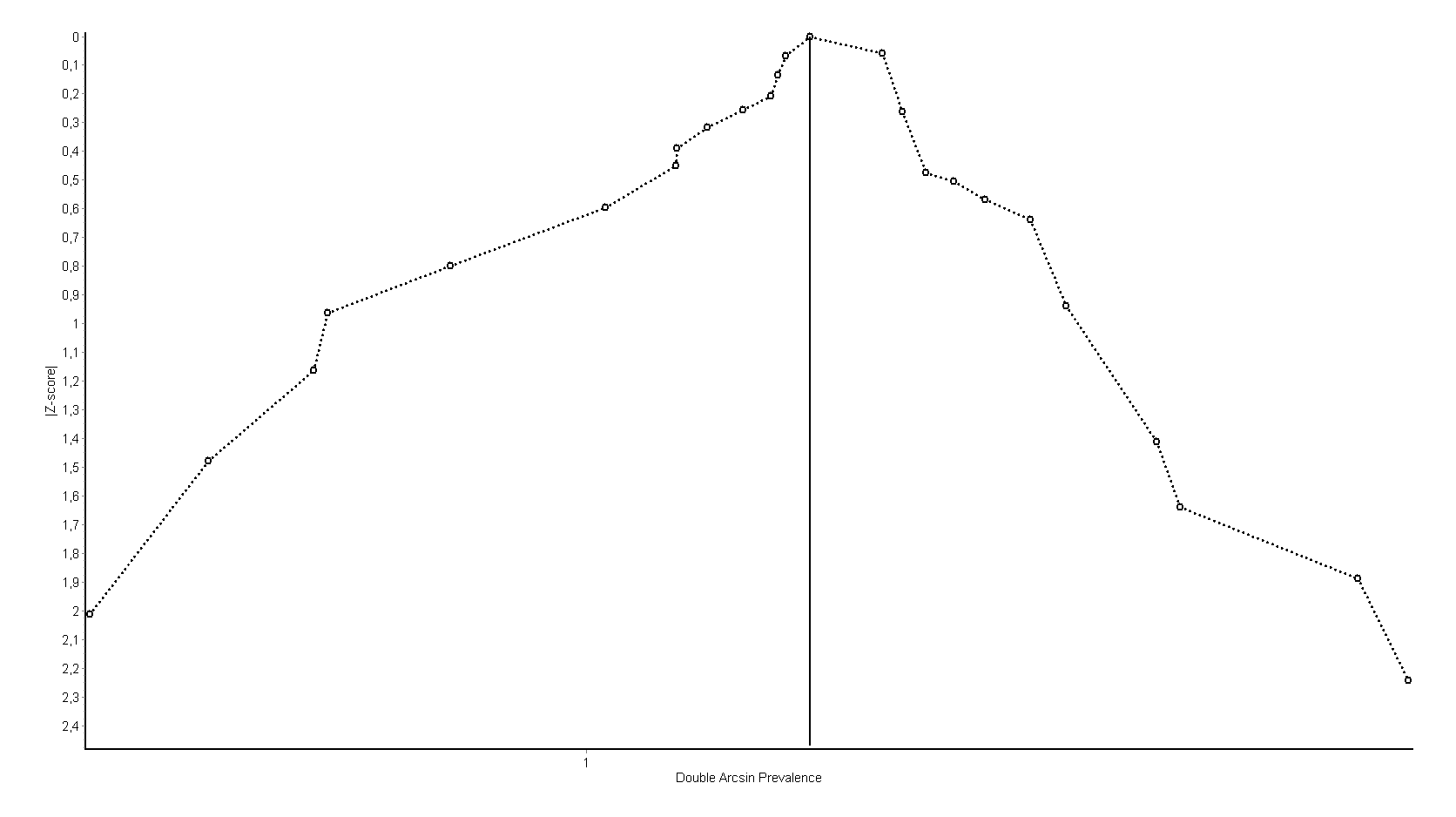

Supplement: Supplementary file 1 [file bjosup.zip › S2056472421000545sup003.tif]

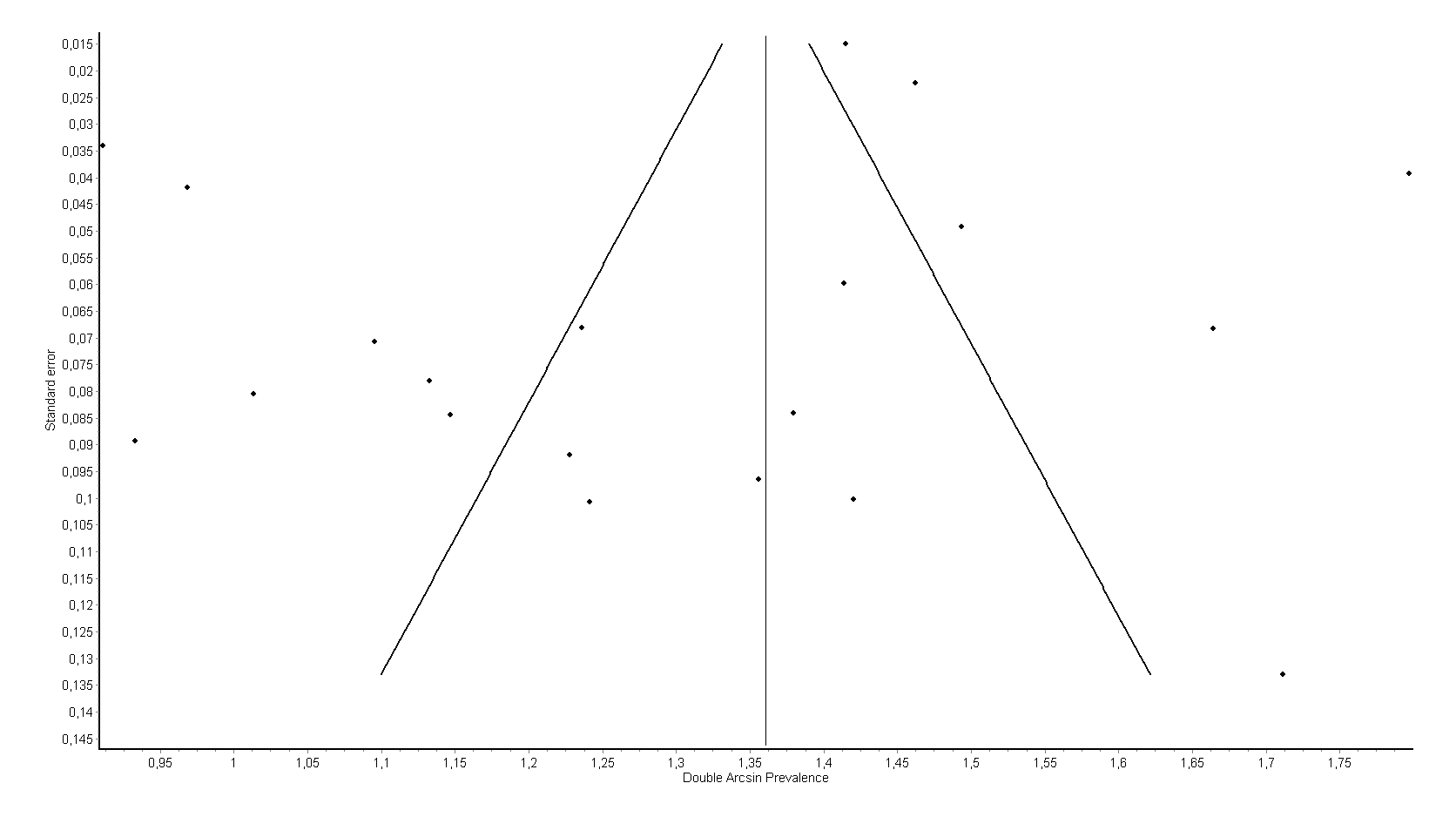

Supplement: Supplementary file 1 [file bjosup.zip › S2056472421000545sup004.tif]

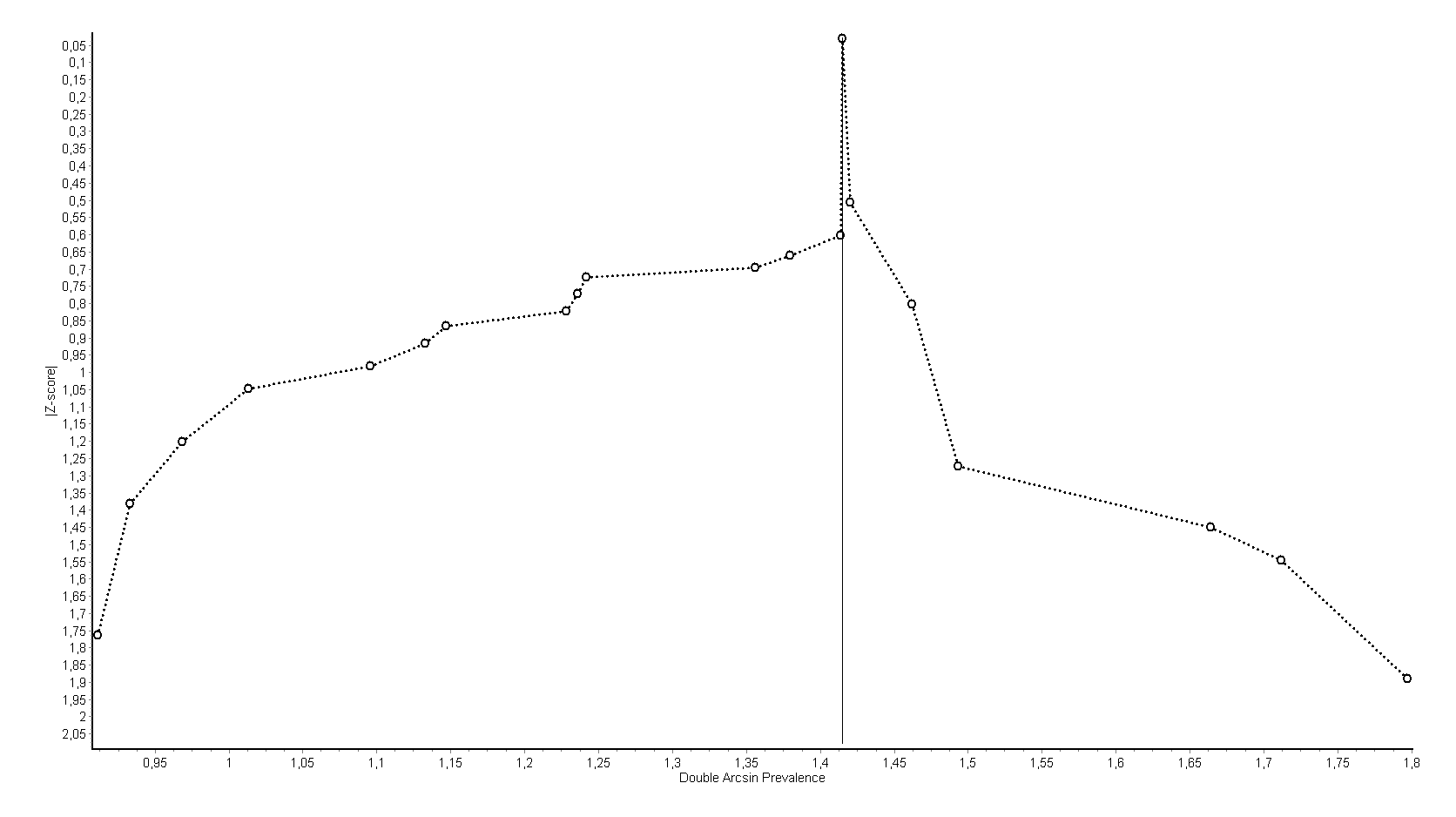

Supplement: Supplementary file 1 [file bjosup.zip › S2056472421000545sup005.tif]
